# Supplementary material for: Accounting for population structure and data quality in demographic inference with linkage disequilibrium methods
Source: Nat Commun. 2025 Jul 1;16:6054. doi: 10.1038/s41467-025-61378-w (PMC12218012; doi:10.1038/s41467-025-61378-w)
Supplement: Supplementary file 1 — Supplementary Information [file 41467_2025_61378_MOESM1_ESM.pdf]

Accounting for Population Structure and Data Quality in Demographic Inference with Linkage Disequilibrium Methods

Enrique Santiago, Carlos Köpke and Armando Caballero

Supplementary Information

(This file contains Supplementary Figures and Tables, and an appendix with Theoretical Derivations)

Supplementary Figures and Tables

Supplementary Figure 1. Partitioning of total metapopulation LD (δ^2) into its within-subpopulation, between-subpopulation, and between-within (i.e. $\delta^2 = \delta_w^2 + \delta_b^2 + 2 \cdot \delta_{bw}^2$) components as a function of the recombination rate c. Lines are predictions using Eq. (1) for a metapopulation composed of two subpopulations of N = 1000 individuals each and a reciprocal migration rate m = 0.001, corresponding to a fixation index F_{ST} = 0.0588. Colored areas represent the size of each component. Observed results from simulations for different recombination rates are indicated by crosses (components) and dots (total LD). A two-locus system was simulated for 7×10^8 consecutive generations for each recombination rate, with random reintroduction of a new mutation each time a fixation or loss of alleles occurs at either locus. The small section within the figure is an enlargement for the highest range of c values. A and B indicate the two sections of the LD spectrum, weakly linked loci vs. unlinked loci, that are compared to infer the parameters of the metapopulation.

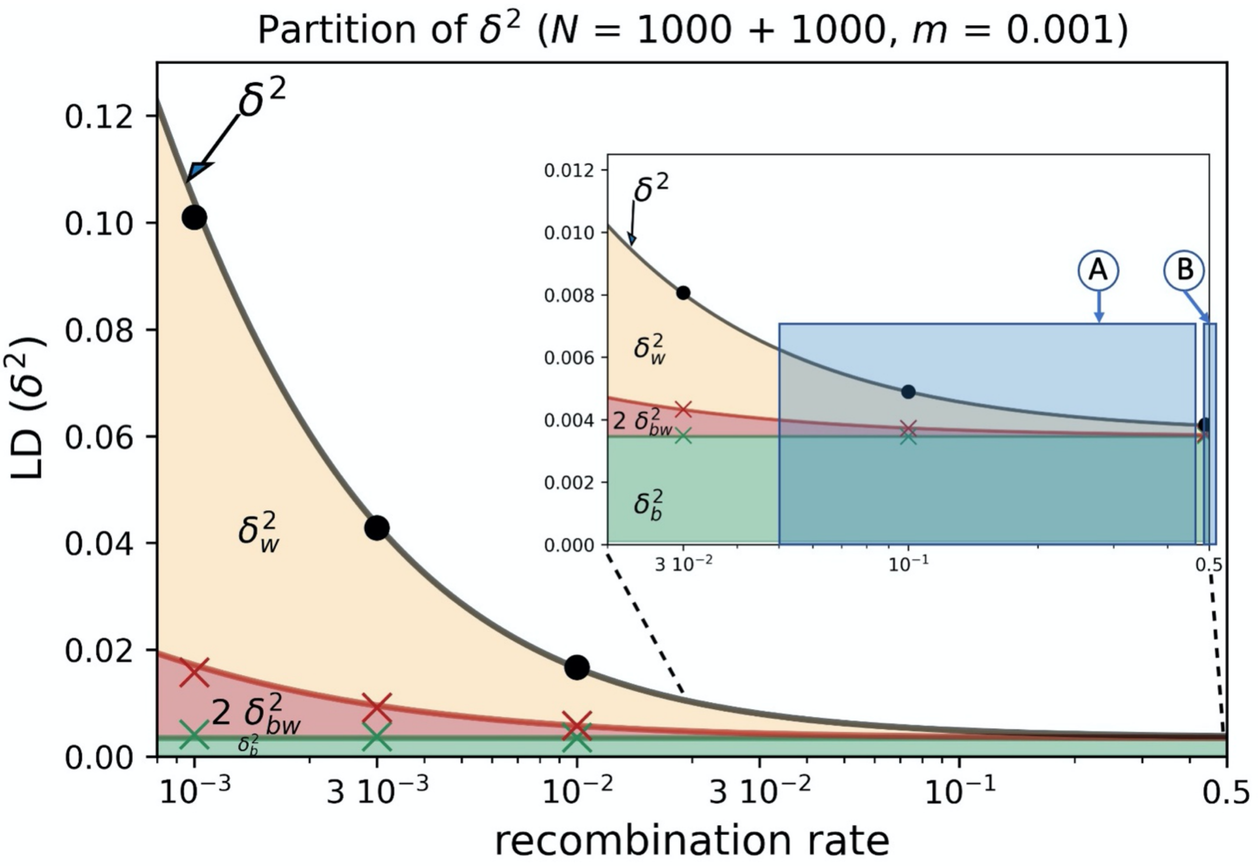

Supplementary Figure 2. *currentNe2* estimates in additional simulated scenarios. The top plot in each panel shows estimates of contemporary effective population size (N_e) for the entire metapopulation (blue dots), the sum of the effective sizes of subpopulations (N_T, red dots), and N_e assuming panmixia (green squares; green arrows indicate values below the scale limit). Note that the labels '*N'* on the x-axes here refer generically to the three estimates. The three estimates are plotted on the same scale *N*. The three lower plots show estimates for migration rates (*m*), genetic differentiation indices (F_{ST}), and the number of subpopulations (*s*). All estimates were calculated under the assumption of population subdivision (option '-x'), except for N_e under panmixia. Each estimate represents the geometric mean from ten simulated metapopulations, with 95% confidence intervals. True metapopulation values are indicated by grey dashed lines (N_e and N_T lines often overlap, with N_e typically higher). A) Effect of differences in subpopulation sizes in an island model, where one subpopulation accounts for 50% of the total metapopulation size, while the remaining subpopulations share the remaining 50% equally. B) Effect of increasing the number of subpopulations in a linear stepping stone model, with equal subpopulation sizes and symmetric migration (m = 0.005). C) Effect of increasing the number of subpopulations in a continent-island model, where a large subpopulation (50% of the total metapopulation size) exchanges migrants with smaller subpopulations of equal size. D) Effect of asymmetric migration between two islands, where migration rates differ between directions (migration pop1→pop2 ≠ migration pop2→pop1). All estimates are based on random samples of 100 individuals from the total metapopulation.

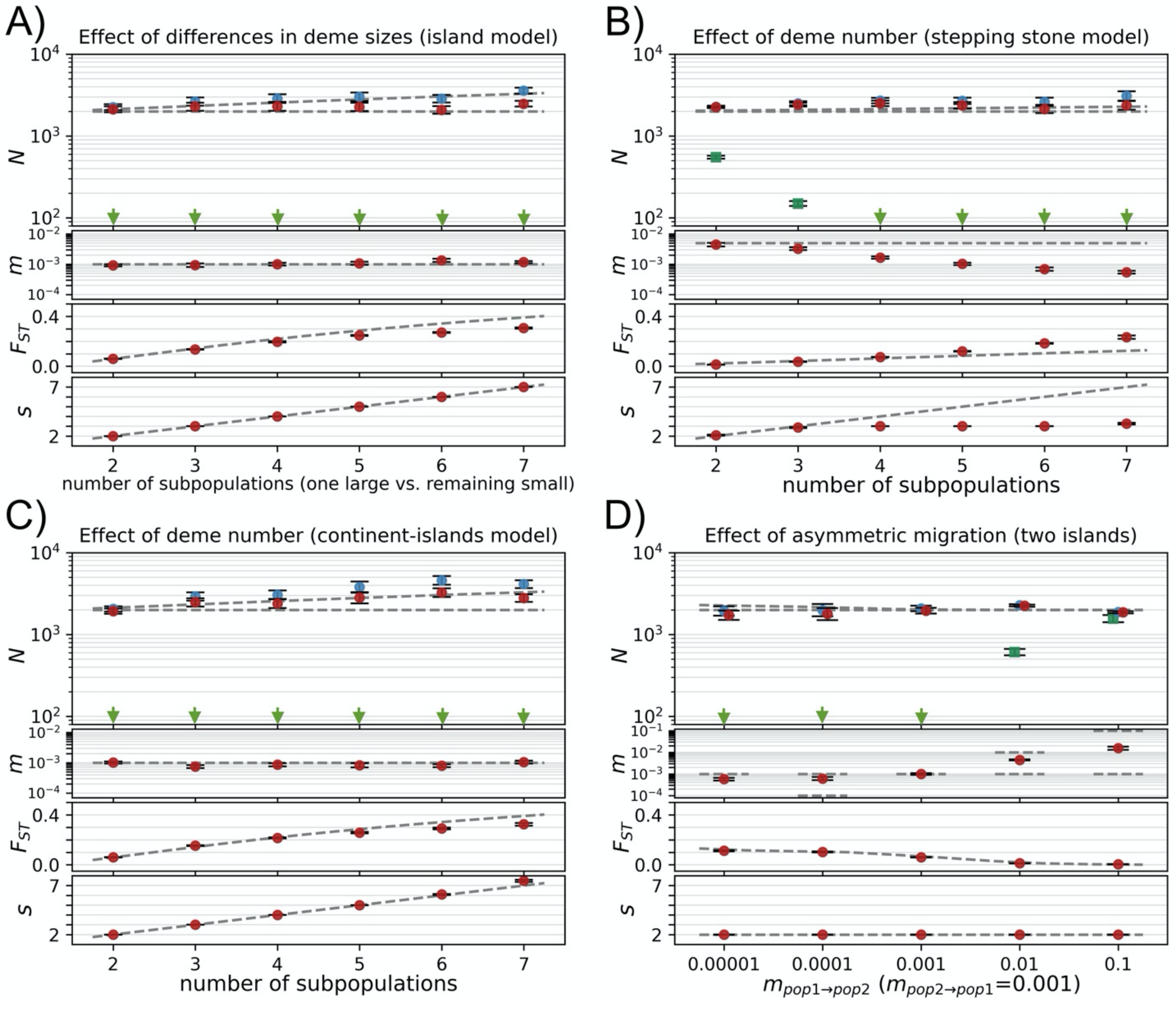

Supplementary Figure 3. A comparison of historical N_T estimates using *GONE2* and *HapNe-LD* under two metapopulation scenarios. Five replicates of genome simulations with 22 autosomes of 250 Mb each, with a uniform recombination rate of 1 cM per Mb, were performed for each scenario. The top panel shows the results for metapopulations consisting of two subpopulations of 1000 individuals each. These subpopulations were maintained with constant size over many generations, with a reciprocal migration rate of 0.001. The bottom panel shows the results for metapopulations consisting of two subpopulations, initially containing 1000 individuals each, that experienced a reduction in population size affecting both subpopulations 30 generations prior to sampling, while maintaining a constant migration rate of 0.001. Analyses were performed using random samples of 100 individuals drawn from the entire metapopulation. The shaded areas around the lines represent the 95% confidence intervals of the distribution of the estimates calculated with the five replicates. Grey areas represent the true metapopulation size. The population size output produced by *HapNe-LD* is haploid, and was divided by 2 to obtain diploid size estimates, as indicated on the software webpage (https://github.com/palamaraLab/hapne).

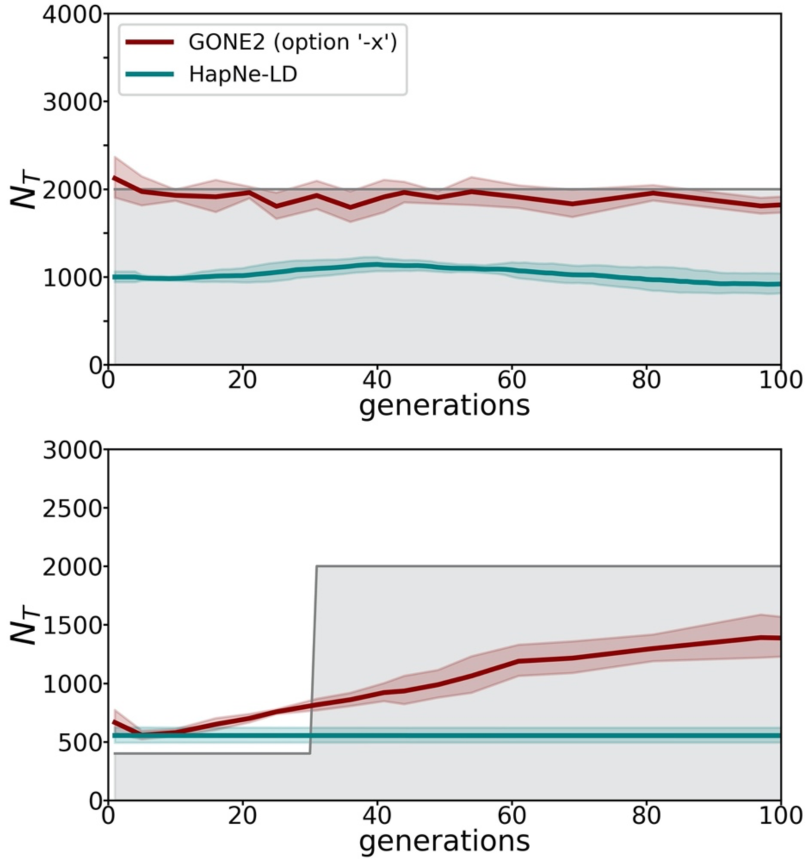

Supplementary Figure 4. Relationship between the estimates of N_e assuming panmixia ($N_{e,pan}$) and assuming population structure ($N_{e,met}$), and the migration rate estimate (m) for the range of species analyzed with *currentNe2* in Table 1 of the main text.

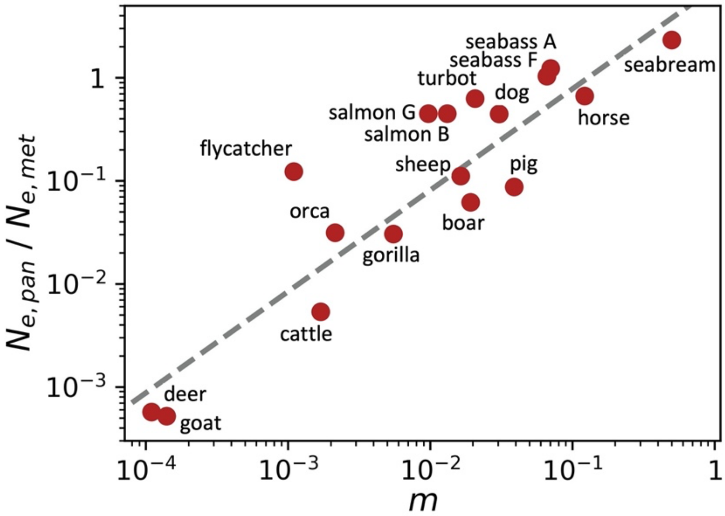

Supplementary Figure 5. Pseudocode describing the inner workings of the algorithm for determining the effective population size under the assumption of population subdivision. The input parameters are the observed LD values between sites in different chromosomes (δ_{05}^2) and between weakly linked sites in the same chromosome (δ_{link}^2), the observed inbreeding coefficient (*f*), the proposed number of subpopulations (*s*) and the sample size. The outputs are the combination of migration rate (*m*), the subpopulation size (*N*), and the number of subpopulations (*s*) that minimise the error. The genetic differentiation index (F_{ST}) is calculated using the equation of Takahata[9]. Finally the effective sizes are calculated as $N_T = sN$ and $N_e = N_T/(1 - F_{ST})$.

procedure APPROXIMATENEFROMFST($\delta_{05}^2, \delta_{link}^2, f, sampleSize$)
// δ_{05}^2 and δ_{link}^2 are computed from the sample
$\delta_{b05}^2 = Getd2Between(\delta_{05}^2, f, sampleSize)$
$best_{F_{ST}} = 0$
$best_m = 0$
$best_s = 0$
$minError = 9999999$
$best_N = 0$
for $s \leftarrow 2, 100$ **do**
$min_{F_{ST}} = 0$
$F_{ST} = min_{F_{ST}}$
$max_{F_{ST}} = \sqrt{\delta_{b05}^2 * (s-1)}$
$delta_{F_{ST}} = (maxF_{ST} - min_{F_{ST}})/10000$
while $F_{ST} < max_{F_{ST}}$ **do**
// Initialize loop variables
$Initialize(N, max_N, logDelta)$
$F_{ST} = F_{ST} + delta_{F_{ST}}$
while $N < max_N$ **do**
$N = N + 10^{logDelta}$
$m = (1 - F_{ST}) \ / \ (4 * N * F_{ST} * (s/(s-1))^2)$
// Compute intra-chromosome δ^2 as described in eq. A13
$\delta_{pred1}^2 = ComputeIntraChromo\_\delta^2(N, F_{ST}, m, s)$
// Compute inter-chromosome δ^2 as described in eq. A13
$\delta_{pred2}^2 = ComputeInterChromo\_\delta^2(N, F_{ST}, m, s)$
$error = ((\delta_{pred1}^2 - \delta_{link}^2)/\delta_{link}^2)^2 + ((\delta_{pred2}^2 - \delta_{05}^2)/\delta_{05}^2)^2$
if $error < minError$ **then**
$best_N = N$
$minError = error$
$best_{F_{ST}} = F_{ST}$
$best_m = m$
$best_s = s$
end if
$N = best_N$
$logDelta = logDelta/50$
end while
$min_{F_{ST}} = best_{F_{ST}} - delta_{F_{ST}}$
$max_{F_{ST}} = best_{F_{ST}} + delta_{F_{ST}}$
$delta_{F_{ST}} = (max_{F_{ST}} - min_{F_{ST}})/10000$
$F_{ST} = min_{F_{ST}}$
end while
end for
end procedure

Supplementary Table 1. Partition of LD in metapopulations with two subpopulations. Simulated (s) and predicted [equation (1), (p)] components of LD ($\delta^2 = \delta_w^2 + 2 \cdot \delta_{bw}^2 + \delta_b^2$) for metapopulations composed of two equally sized subpopulations. A two-locus system was simulated for 7 $\times 10^8$ consecutive generations for each combination of subpopulation size (*N*), migration rate (*m*), and recombination rate (*c*), with random reintroduction of a new mutation each time a fixation or loss of alleles occurs at either locus.

N	*m*	*c*	F_{ST}	δ_w^2 (s)	δ_w^2 (p)	δ_{bw}^2 (s)	δ_{bw}^2 (p)	δ_b^2 (s)	δ_b^2 (p)	δ^2 (s)	δ^2 (p)
1000	0.000001	0.001	0.98425	0.0005882	0.0000243	0.0017471	0.0025814	0.9739972	0.9687520	0.9780719	0.9739390
1000	0.000001	0.01	0.98425	0.0000249	0.0000030	0.0003391	0.0003692	0.9696762	0.9687520	0.9703801	0.9694933
1000	0.000001	0.5	0.98425	0.0000001	0.0000001	0.0000077	0.0000077	0.9690320	0.9687520	0.9690475	0.9687675
1000	0.000010	0.001	0.86207	0.0061260	0.0018667	0.0138247	0.0196124	0.7782168	0.7431650	0.8121398	0.7842564
1000	0.000010	0.01	0.86207	0.0003982	0.0002327	0.0026449	0.0028272	0.7512944	0.7431650	0.7569379	0.7490521
1000	0.000010	0.5	0.86207	0.0000065	0.0000063	0.0000586	0.0000594	0.7440023	0.7431650	0.7441276	0.7432901
1000	0.000100	0.001	0.38464	0.0448346	0.0371550	0.0284292	0.0360448	0.1722339	0.1479472	0.2748884	0.2571917
1000	0.000100	0.01	0.38464	0.0049642	0.0046317	0.0051821	0.0055380	0.1505294	0.1479472	0.1658477	0.1636549
1000	0.000100	0.5	0.38464	0.0001273	0.0001262	0.0001173	0.0001183	0.1473451	0.1479472	0.1477079	0.1483099
1000	0.001000	0.001	0.05888	0.0852172	0.0869058	0.0058210	0.0064738	0.0041550	0.0034667	0.1010142	0.1033201
1000	0.001000	0.01	0.05888	0.0108938	0.0108335	0.0011003	0.0011433	0.0036163	0.0034667	0.0167106	0.0165869
1000	0.001000	0.5	0.05888	0.0002954	0.0002952	0.0000276	0.0000276	0.0034985	0.0034667	0.0038491	0.0038172
1000	0.010000	0.001	0.00627	0.0935017	0.0968927	0.0006076	0.0006271	0.0000479	0.0000394	0.0947648	0.0981862
1000	0.010000	0.01	0.00627	0.0120075	0.0120785	0.0000893	0.0000912	0.0000412	0.0000394	0.0122273	0.0123003
1000	0.010000	0.5	0.00627	0.0003296	0.0003291	0.0000030	0.0000031	0.0000402	0.0000394	0.0003757	0.0003746
100	0.010000	0.001	0.05938	0.2936333	0.2953929	0.0181959	0.0192679	0.0057923	0.0035263	0.3358174	0.3374550
100	0.010000	0.01	0.05938	0.0862475	0.0874597	0.0058647	0.0065628	0.0042660	0.0035263	0.1022429	0.1041115
100	0.010000	0.5	0.05938	0.0029697	0.0029438	0.0002625	0.0002735	0.0035915	0.0035263	0.0070863	0.0070171
10,000	0.000100	0.001	0.05883	0.0109408	0.0107820	0.0011385	0.0011406	0.0036457	0.0034609	0.0168635	0.0165241
10,000	0.000100	0.01	0.05883	0.0011103	0.0011098	0.0001378	0.0001351	0.0035527	0.0034609	0.0049386	0.0048409
10,000	0.000100	0.5	0.05883	0.0000296	0.0000295	0.0000027	0.0000028	0.0034630	0.0034609	0.0034980	0.0034959

Supplementary Table 2. Partition of LD in metapopulations with four subpopulations. Simulated (s) and predicted [equation (1), (p)] components of LD ($\delta^2 = \delta_w^2 + 2 \cdot \delta_{bw}^2 + \delta_b^2$) for metapopulations composed of four equally sized subpopulations. A two-locus system was simulated for 7 ×10[8] consecutive generations for each combination of subpopulation size (N_{subp}), migration rate (m), and recombination rate (c), with random reintroduction of a new mutation each time a fixation or loss of alleles occurs at either locus.

N_{subp}	m	c	F_{ST}	δ_w^2 (s)	δ_w^2 (p)	δ_{bw}^2 (s)	δ_{bw}^2 (p)	δ_b^2 (s)	δ_b^2 (p)	δ^2 (s)	δ^2 (p)
500	0.000001	0.001	0.99646	0.0001208	0.0000012	0.0003716	0.0003918	0.4070272	0.3309755	0.4078912	0.3317603
500	0.000001	0.01	0.99646	0.0000086	0.0000002	0.0000742	0.0000560	0.3453467	0.3309755	0.3455037	0.3310878
500	0.000001	0.5	0.99646	0.0000000	0.0000000	0.0000016	0.0000012	0.3111131	0.3309755	0.3111164	0.3309779
500	0.000010	0.001	0.96567	0.0012494	0.0001157	0.0036104	0.0036421	0.3882403	0.3108366	0.3967105	0.3182364
500	0.000010	0.01	0.96567	0.0000882	0.0000144	0.0006800	0.0005253	0.3223089	0.3108366	0.3237569	0.3119016
500	0.000010	0.5	0.96567	0.0000005	0.0000004	0.0000166	0.0000110	0.3116610	0.3108366	0.3116946	0.3108591
500	0.000100	0.001	0.73772	0.0131803	0.0067499	0.0218979	0.0193846	0.2408136	0.1814092	0.2977896	0.2269283
500	0.000100	0.01	0.73772	0.0012605	0.0008414	0.0039814	0.0030151	0.1905302	0.1814092	0.1997536	0.1882809
500	0.000100	0.5	0.73772	0.0000233	0.0000229	0.0000956	0.0000644	0.1804912	0.1814092	0.1807056	0.1815609
500	0.001000	0.001	0.21963	0.0632929	0.0597534	0.0182326	0.0107199	0.0227377	0.0160786	0.1224958	0.0972718
500	0.001000	0.01	0.21963	0.0078325	0.0074488	0.0033489	0.0023130	0.0172625	0.0160786	0.0317927	0.0281534
500	0.001000	0.5	0.21963	0.0002036	0.0002030	0.0000852	0.0000569	0.0161945	0.0160786	0.0165684	0.0163953
500	0.010000	0.001	0.02753	0.0898562	0.0927911	0.0026199	0.0009947	0.0003578	0.0002527	0.0954539	0.0950332
500	0.010000	0.01	0.02753	0.0115548	0.0115672	0.0003989	0.0001974	0.0002723	0.0002527	0.0126250	0.0122147
500	0.010000	0.5	0.02753	0.0003159	0.0003152	0.0000128	0.0000086	0.0002560	0.0002527	0.0005974	0.0005850
50	0.010000	0.001	0.22066	0.2075587	0.2027811	0.0548438	0.0258403	0.0357829	0.0162303	0.3530292	0.2706920
50	0.010000	0.01	0.22066	0.0632026	0.0600393	0.0179867	0.0106922	0.0226706	0.0162303	0.1218466	0.0976539
50	0.010000	0.5	0.22066	0.0020867	0.0020209	0.0008056	0.0005464	0.0163839	0.0162303	0.0200816	0.0193440
5000	0.000100	0.001	0.21952	0.0077924	0.0074145	0.0033466	0.0023130	0.0172157	0.0160635	0.0317012	0.0281040
5000	0.000100	0.01	0.21952	0.0007676	0.0007632	0.0004191	0.0002786	0.0163541	0.0160635	0.0179600	0.0173840
5000	0.000100	0.5	0.21952	0.0000203	0.0000203	0.0000084	0.0000057	0.0160695	0.0160635	0.0161066	0.0160953

Supplementary Table 3. Estimates of N_e using *currentNe* in panmictic and subdivided populations. N_e estimates and their upper and lower 90 % confidence limits (CL) from simulation data assuming either a single panmictic population of size $N = 2000$ diploid individuals (panmictic population) or a subdivided population with two subpopulations of size $N = 1000$ and migration rate of $m = 0.001$ per generation, under different demographic scenarios. Estimates ignore migration or take into account switching off and on the option –x. Constant: Unchanged population size of $N = 2000$ diploid individuals. Drop: A population of size $N = 2000$ individuals (two of 1000 in the subdivided scenarios) suddenly drops to $N = 200$ (two of 100 in the subdivided scenario) at generation 50 back in time. Decline and recovery: A population of size $N = 2000$ individuals (two of 1000 in the subdivided scenarios) suddenly declines to $N = 200$ (two of 100 in the subdivided scenarios) at generation 50 back in past and immediately recovers to its initial time at generation 30 back in the past. Growth: A population of size $N = 2000$ individuals (two of 1000 in the subdivided scenarios) grows linearly to $N = 20{,}000$ (two of 10,000 in the subdivided scenarios) in the last 50 generations. The simulations were performed using the SLiM3 software for 10,000 generations assuming 20 chromosomes of 100 Mb and 100 cM each. The results are the averages of 10 replicates of each scenario analysing approximately 40,000 SNPS in the last generation from a sample of n = 100 individuals. Estimates of N_e assuming migration that are significantly larger than the corresponding estimates without migration are shown in red.

.

Population demography	Current census	Estimates ignoring migration N_e	Upp CL	Low CL	Estimates assuming migration N_T	Upp CL	Low CL
Panmictic population							
Constant	2,000	2,117	1,578	2,838	2,300	1,699	3,112
Drop	200	104	95	115	116	105	128
Drop and recovery	2,000	1,568	1,203	2,042	2,139	1,587	2,882
Growth	20,000	9,459	5,459	16,390	24,199	10,961	53,424
Subdivided population (n = 2)							
Constant	1000 × 2	31	30	33	2,189	1,627	2,944
Drop	100 × 2	5	5	5	368	316	427
Drop and recovery	1000 × 2	12	12	12	1,402	1,089	1,805
Growth	10,000 × 2	42	40	45	5,385	3,534	8,208

Supplementary Table 4. Estimates of N_e using *currentNe* under different degrees of subpopulation differentiation. Estimates of contemporary effective population size and their upper and lower 90 % confidence limits (CL) obtained from simulation data assuming a subdivided population with five subpopulations of size N = 400 individuals (total population size N_T = 2000) or N = 2000 individuals (total population size N_T = 10,000) and migration rate m per generation. Estimates ignore migration or allow for it with the option –x. The last column shows the estimated migration rate (m) by the software. The simulations were run with the software SLiM3 for 10,000 generations assuming 20 chromosomes of 100 Mb and 100 cM each. The results are the averages of 10 replicates of each scenario analysing around 40,000 SNPS in the most recent generation from a sample of n = 100 individuals. Estimates of N_e assuming migration significantly larger than the corresponding ones ignoring migration are shown in red.

Migration m	*Nm*	Estimates ignoring migration			Estimates assuming migration			
		N_e	Upp CL	Low CL	N_T	Upp CL	Low CL	*Est. m*
5 subpops of N = 400; N_T = 2000								
0.0001	0.04	3	3	5	2,505	1,811	3,465	0.00011
0.0005	0.2	4	4	5	1,756	1,341	2,301	0.00062
0.001	0.4	8	8	8	2,285	1,691	3,089	0.00090
0.005	2	83	76	89	2,576	1,879	3,532	0.00393
0.01	4	281	247	321	2,420	1,778	3,293	0.00797
0.025	10	919	744	1,135	2,286	1,690	3,093	0.01465
0.05	20	1,544	1,192	2,001	2,214	1,643	2,982	0.01701
0.1	40	1,859	1,407	2,456	2,284	1,689	3,089	0.01386
0.15	60	1,988	1,493	2,646	2,265	1,677	3,060	0.01348
5 subpops of N = 2000; N_T = 10,000								
0.00005	0.1	4	4	5	22,069	10,121	48,124	0.00005
0.0001	0.2	6	5	6	21,446	9,994	46,020	0.00007
0.0005	1	31	29	32	13,541	7,312	25,076	0.00040
0.001	2	92	85	101	12,172	6,755	21,935	0.00086
0.002	4	327	283	377	13,739	7,418	25,446	0.00149
0.005	10	1,748	1,325	2,306	12,453	6,866	22,586	0.00416
0.01	20	5,171	3,440	7,773	12,741	7,153	22,697	0.00691
0.02	40	7,723	4,788	12,457	11,436	6,573	19,895	0.00771
0.05	60	10,039	5,905	17,054	11,882	6,773	20,842	0.01123

Theoretical Derivations

Contents:

1- Panmictic Populations.
  1.1- LD in Haploids with Constant Population Size.
  1.2- LD in Diploids with Constant Population Size.
  1.3- The Contribution of New Mutations.
  1.4- LD in Haploids with Variable Population Size.
  1.5- LD in Diploids with Variable Population Size.
  1.6- Correction for Sampling Haploids.
  1.7- Correction for Sampling Diploids.
  1.8- Correction for Sampling Diploids with Unknown Phase.
  1.9- Correction for Low-Coverage Sequencing (Pseudo-Haploids).
  1.10- Correction for Base-Calling Errors.
2- Subdivided Populations (Islands Model).
  2.1- LD Partition.
  2.2- Expectations of LD Components for Haploids and Diploids.
  2.3- Correction for Sampling Haploids and Diploids.
  2.4- Correction for Sampling Diploids with Unknown Phase
  2.5- Correction for Pseudo-Haploids.
3- Some Statistical Equations.
  3.1- Expectation of the Sample Product of Two Variances.
  3.2- Expectation of the Square of the Sample Covariance.
  3.3- Expectation for the Sample Moment m_{22}.
  3.4- Approximation to the Population Moment μ_{22} at Equilibrium.
4- Estimation of the Inbreeding Coefficient.
5- Supplementary References.

(Parts of the derivation from Santiago et al. (2020)[1] are repeated here for clarity)

1- Panmictic Populations.

This section examines the evolution of linkage disequilibrium (LD) between two genomic sites in a single panmictic population model with discrete generations and random mating. LD is defined as the ratio of expectations $\delta^2 = \frac{E[D^2]}{E[W]}$ by Ohta and Kimura[2], where D^2 represents the squared covariance between allelic values at the two sites within haplotypes, and W is the product of the genic variances of the two sites.

1.1- LD in Haploids with Constant Population Size.

The population consists of 2*N* haploid parents mating randomly to produce 2*N* haploid offspring. Let *c* be the recombination rate between the two polymorphic sites *X* and *Y*, *t* the current generation and D_t^2 the squared covariance of allele values between sites in haploid genomes at generation *t* (point 1 in Supplementary Figure 6). The expected value of the squared covariance in the next generation D_{t+1}^2 can be approximated in two steps: first, by considering the effect of recombination, and then by considering the sampling process.

Recombination changes the expectation of the squared covariance from D_t^2 to $D_t'^2$ in the infinite pool of gametes (point 2 in Supplementary Figure 6). The expectation is given by the following equation, where x_i and y_i represent the allele values at sites *X* and *Y*, respectively, in individual *i* at generation *t*:

$$D_t'^2 = \left[\frac{x_1y_1(1-c) + x_2y_2(1-c) + \cdots + x_{2N}y_{2N}(1-c) + x_1y_1\frac{c}{2N} + x_1y_2\frac{c}{2N} + \ldots x_2y_1\frac{c}{2N} + x_1y_2\frac{c}{2N} + \cdots}{2N}\right]^2$$

$$= \left[(1-c)\frac{\sum_{i=1}^{2N} x_iy_i}{2N} + \frac{c}{2N}\frac{\sum_{i=1}^{2N}\sum_{j=1}^{2N} x_iy_j}{2N}\right]^2$$

Here, x_i and y_i are deviations from the population mean value. By expanding the equation, we get:

$$D_t'^2 = (1-c)^2\left[\frac{\sum_{i=1}^{2N} x_iy_i}{2N}\right]^2 + c^2\left[\frac{\sum_{i=1}^{2N}\sum_{j=1}^{2N} x_iy_j}{4N^2}\right]^2 + 2c(1-c)\cdot\frac{\sum_{i=1}^{2N} x_iy_i}{2N}\cdot\frac{\sum_{i=1}^{2N}\sum_{j=1}^{2N} x_iy_j}{4N^2}$$

The first term on the right hand side represents the remaining part of the original squared covariance D_t^2 after recombination. The second term, which is the sum of all possible products of the allele values at the two sites, equals zero, i.e. $E[x_iy_jx_ky_l] = 0$ for arbitrary subscripts i, j, *k* and *l* between 1 and 2*N*. The third term, representing the sum of all the cross-products of the allelic values of the parental and the recombinant gametes, has a marginal value under random mating, i.e. $E[x_iy_ix_ky_l] \approx 0$. Consequently, we obtain the simplified expression:

$$D_t'^2 \approx (1-c)^2\, D_t^2 \quad \text{(A1)}$$

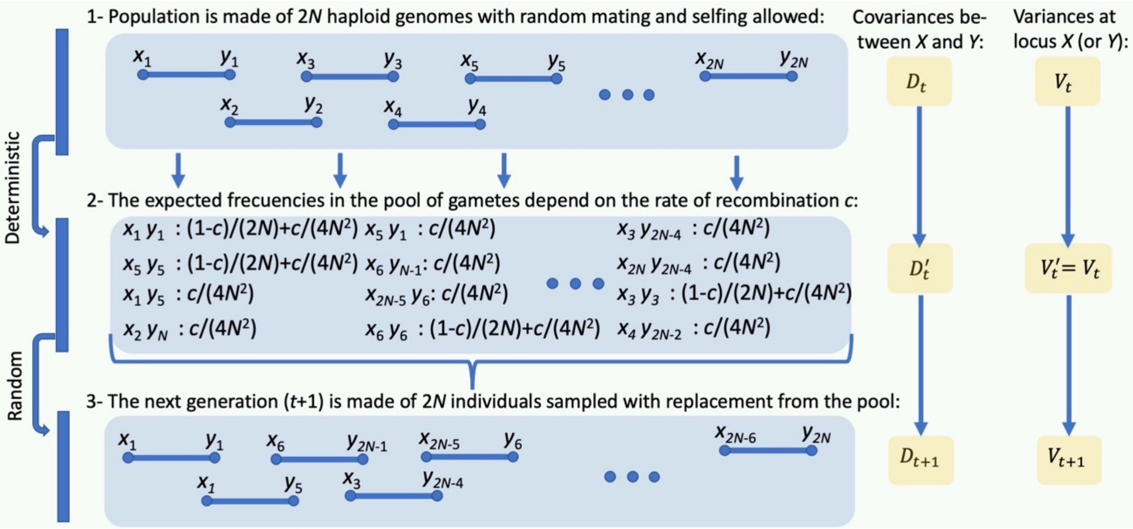

Supplementary Figure 6. Process of transitioning from one generation to the next one in a haploid population.

After random sampling of $2N$ gametes (point 3 in Supplementary Figure 6), the expected D_{t+1}^2 is the sum of the remaining $D_t'^2$ reduced by sampling and the increase in covariance due to drift acting on the standing variation (see derivation of eq. A19 in Section 3.2):

$$D_{t+1}^2 \approx D_t^{2\prime}\left(1-\frac{2.2}{2N}\right)+W\frac{1}{2N} \tag{A2}$$

where W is the product of the genetic variances V_xV_y at both loci. This equation provides a good approximation for systems where most of LD at equilibrium has been generated in distant generations, i.e. when N is large and c is small. Otherwise the factor 2.2 increases slightly but becomes irrelevant because $W \gg D_t'^2$, especially when c is large.

Substituting $D_t'^2$ by its value given in eq. A1:

$$D_{t+1}^2 = (1-c)^2\, D_t^2\left(1-\frac{2.2}{2N}\right)+W\frac{1}{2N} \tag{A3}$$

Ignoring mutations, D^2 at equilibrium equals the sum of the remaining D^2 after recombination and the increase in D^2 due to drift, i.e.

$$D^2 = (1-c)^2 D^2\left(1-\frac{2.2}{2N}\right)+W\frac{1}{2N}$$

Dividing both sides by W, which remains invariant at equilibrium, we obtain the expectation of LD:

$$\delta_c^2 = \frac{D^2}{W} = \frac{1}{2N(1-(1-c)^2)+2.2(1-c)^2} \tag{A4}$$

1.2- LD in Diploids with Constant Population Size.

The population consists of $2N$ haploid genomes randomly arranged into N diploid monoecious individuals. Each offspring is generated by a new random pairing. In diploids, the specific pairing of haploid genomes in parents at generation t (point 1 in Supplementary Figure 7) determines the expectation of gametes (point 2 in Supplementary Figure 7), since recombination occurs only within individuals. Recombination events produce new combinations of alleles, changing the squared covariance in the infinite pool of gametes to $D_t'^2$. The expectation is given by the following equation, where x_i and y_i represent the allele values at sites X and Y, respectively, in genome i at generation t.

$$D_t'^2 = \left[\frac{x_1y_1\frac{(1-c)}{2} + x_2y_2\frac{(1-c)}{2} + x_1y_2\frac{c}{2} + x_2y_1\frac{c}{2} + \cdots + x_{2N-1}y_{2N-1}\frac{(1-c)}{2} + x_{2N}y_{2N}\frac{(1-c)}{2} + x_{2N-1}y_{2N}\frac{c}{2} + x_{2N}y_{2N-1}\frac{c}{2}}{N}\right]^2$$

$$= \left[\frac{(1-c)}{2}\frac{\sum_{i=1}^{2N} x_iy_i}{N} + \frac{c}{2}\frac{\sum_{i=1}^{2N} x_iy_j}{N}\right]^2 \quad \text{(A5)}$$

where, x_i and y_i represent deviations from the population mean values. When the subscripts in a product of allele values are equal (e.g., x_iy_i), it refers to the original alleles in a given haploid genome of a diploid individual of generation t, indicating alleles in a non-recombinant gamete produced by that individual. Conversely, when the subscripts are different (e.g., x_iy_j), it represents the product the values of alleles in a recombinant gamete produced by that individual.

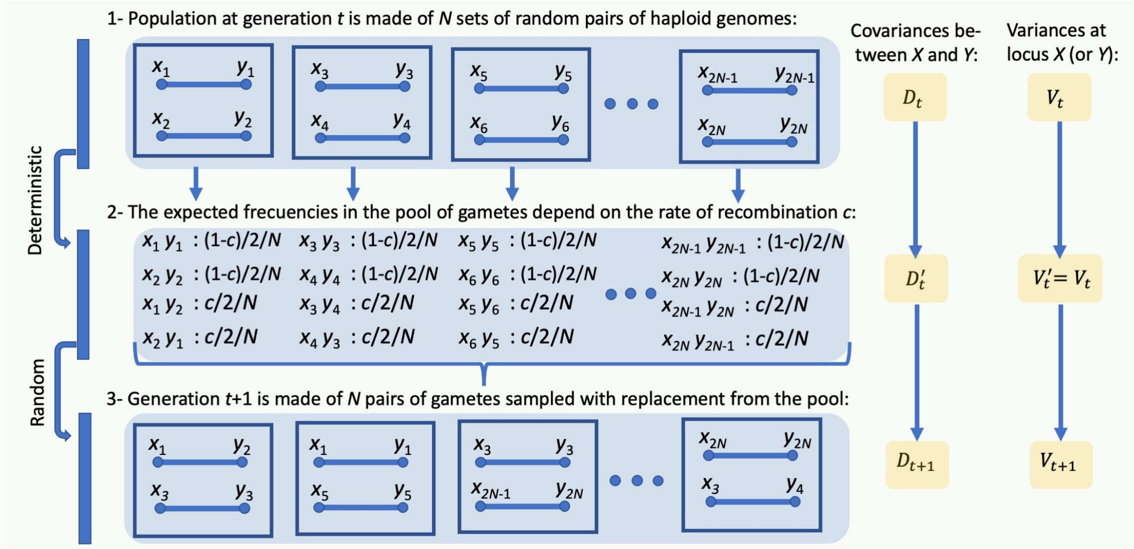

Supplementary Figure 7. Process of transitioning from one generation to the next in a diploid population.

.

Expanding the equation yields:

$$D_t'^2 = (1-c)^2 \left[\frac{\sum_{i=1}^{2N} x_i y_i}{2N}\right]^2 + c^2 \left[\frac{\sum_{i=1}^{2N} x_i y_j}{2N}\right]^2 + 2c(1-c)\frac{\sum_{i=1}^{2N} x_i y_i}{2N}\frac{\sum_{l=1}^{2N} x_l y_k}{2N}$$

The first term on the right-hand side represents the remaining part of the original squared covariance D_t^2 after recombination. The third term represents the sum of the cross-products of the allelic values of gametes of different individuals, and has a marginal value under random mating. This leads to the simplification:

$$D_t'^2 \approx (1-c)^2\, D_t^2 + c^2 \left[\frac{\sum_{i=1}^{2N} x_i y_j}{2N}\right]^2 \tag{A6}$$

The second term, which is absent in haploid models with random mating, becomes significant when haploid genomes are paired within diploid individuals, i.e. when recombination is restricted to fixed pairs of haploid genomes. Its expansion is:

$$c^2 \left[\frac{\sum_{i=1}^{2N} x_i y_j}{2N}\right]^2 = c^2 \frac{\sum_{i=1}^{2N} x_i^2 y_j^2}{4N^2} + c^2 \frac{\sum_{i\neq j}^{2N} x_i y_j x_j y_i}{4N^2} + c^2 \frac{\sum_{i\neq j}^{2N} \sum_{i\neq j\neq k\neq l}^{2N} x_i y_j x_k y_l}{4N^2}$$

In the first term on the right, the expectation of $x_i^2 y_j^2$ is equal to the product of variances $W = V_x V_y$, which is assumed to be constant throughout the process. In the second term, the expectation of $x_i y_j x_j y_i$ is effectively D_t^2, with only a small difference due to autocorrelation. The summands in the third term cancel each other under the conditions of random mating and non-overlapping generations. Therefore, the equation for $D_t'^2$ simplifies to:

$$D_t'^2 \approx (1-c)^2\, D_t^2 + c^2 \frac{W}{2N} + c^2 \frac{D_t^2}{2N}$$

Since D_t^2 is much smaller than W at equilibrium, except for tight linkage, the third term becomes irrelevant. The equation reduces to:

$$D_t'^2 \approx (1-c)^2\, D_t^2 + c^2 \frac{W}{2N} \tag{A7}$$

This equation is the diploid version of the equation eq. A1 for haploids. The expected value of D_{t+1}^2 after random sampling of $2N$ gametes (point 3 in Supplementary Figure 7) is the sum of the remaining $D_t'^2$ after reduction by sampling, and the increase in covariance due to drift acting on standing variation (see eq. A19):

$$D_{t+1}^2 \approx D_t^{2\prime}\left(1 - \frac{2.2}{2N}\right) + W\frac{1}{2N}$$

Substituting $D_t'^2$ by its value using eq. A7:

$$\begin{aligned} D_{t+1}^2 &= \left[(1-c)^2\, D_t^2 + c^2 \frac{W}{2N}\right]\left(1 - \frac{2.2}{2N}\right) + W\frac{1}{2N} \\ &\approx (1-c)^2\, D_t^2 \left(1 - \frac{2.2}{2N}\right) + W\frac{c^2}{2N} + W\frac{1}{2N} \end{aligned} \tag{A8}$$

Ignoring the effect of mutations, the equilibrium D^2 equals the sum of the remaining D^2 after recombination and the increase in D^2 due to drift:

$$D^2 = (1-c)^2 D^2 \left(1 - \frac{2.2}{2N}\right) + W\left(\frac{1}{2N} + \frac{c^2}{2N}\right)$$

(A9)

Dividing both sides of the equation by W and rearranging, we get the expectation of LD:

$$\delta_c^2 = \frac{D^2}{W} = \frac{1+c^2}{2N(1-(1-c)^2) + 2.2(1-c)^2}$$

(A10)

The difference between this equation and eq. A4 for haploids is the term c^2 in the numerator. The expectation of LD in diploids is increased because recombination is restricted to fixed pairs of haplotypes, resulting in a higher probability of identical combinations of alleles in recombinant gametes compared to the haploid model. This probability is further increased when the parents are arranged in lifetime pairings, such as monogamy, because full siblings, which are common in these mating systems, can also produce recombinant gametes of the same type[3].

1.3- The Contribution of New Mutations.

Consider a diploid population in mutation-drift equilibrium where *Y* is a monomorphic site in a large genome, with mutation events per site occurring at a very low rate $\mu \ll 1/2N$. when a new mutation occurs at site *Y*, the genetic variance induced at that site and the expected squared covariance with another polymorphic site *X* are:

$$V_y = \frac{1}{2N} \cdot \frac{2N-1}{2N} \approx \frac{1}{2N}$$

$$E[D_{XY}^2] = E\left[p_x\left(\frac{1-p_x}{2N}\right)^2 + (1-p_x)\left(-\frac{p_x}{2N}\right)^2\right] = \frac{V_x}{4N^2} = \frac{W_{XY}}{2N}$$

where p_x is the frequency of the reference allele at site *X* , V_x is the genetic variance at site *X*, and W_{XY} is the product of the genetic variances at both sites.

As site *Y* mutates with a probability $2N\mu$ for the entire population (assuming μ is much smaller than N^{-1}, the expected increase in D^2 due to new mutations is:

$$E[\Delta D_{XY}^2] = 2N\mu \cdot 2 \cdot \frac{E[V_x]}{4N^2}$$

The factor "2" is included because the creation of a new polymorphic site in a genetic system with a number of previously polymorphic sites generates new covariances at twice that number. The increase in D^2 is very small compared to the product of the genetic variances W of pairs of sites at equilibrium:

$$E[W] = E\left[{V_x}^2\right] = 2N\mu \cdot E[V_x]$$

Therefore,

$$\frac{E[\Delta D_{XY}^2]}{E[W]} = \frac{1}{2N^2}$$

Now, including the increment of the squared covariance due to mutation in eq. A9, at equilibrium:

$$D^2 = (1-c)^2 D^2 \left(1 - \frac{2.2}{2N}\right) + W\frac{1+c^2}{2N} + E[\Delta D_{XY}^2]$$

Thus, D^2 at equilibrium is equal to the sum of the fraction of D^2 remaining after recombination and the increase in D^2 due to drift and mutation. Dividing both sides by W and rearranging:

$$\delta_c^2 = \frac{1 + c^2 + 1/N}{2N(1-(1-c)^2) + 2.2(1-c)^2}$$

The derivation for haploids is equivalent, with the result:

$$\delta_c^2 = \frac{1 + 1/N}{2N(1-(1-c)^2) + 2.2(1-c)^2}$$

Therefore, the small factor $1/N$ in the numerator represents the contribution of new mutations to LD. This contribution is negligible and will be ignored in the following analysis.

1.4- LD in Haploids with Variable Population Size.

Consider a random mating population with $2N$ haploid genomes. Since the mutation rate per site is much smaller than $(2N)^{-1}$, most sites are monomorphic, and new mutations occur as singletons. Now, imagine an infinite number of replicates of this population, all evolving under the same conditions. We randomly select polymorphic sites, referred to as sites X. For each site X, consider other sites Y with recombination rate c relative to the polymorphic site. All sites Y are monomorphic in a generation of reference. After new mutations occur in this generation, the genetic variance at the newly mutated sites Y is

$$V_y = \frac{1}{2N}\cdot\frac{2N-1}{2N} \approx \frac{1}{2N}$$

The average variance at polymorphic sites X is denoted as V_x, which has a constant expectation over time. We define W_m as the expectation of the product of variances of both loci, given by $W_m = V_x V_y \approx V_x/(2N)$ across the set of replicates where the new mutation occurs at site Y.

The expected squared covariance between sites X and Y contributed by the new mutations at site Y is:

$$E[D_m^2] = E\left[p\left(\frac{1-p}{2N}\right)^2 + (1-p)\left(-\frac{p}{2N}\right)^2\right] = \frac{V_x}{2N}\cdot\frac{1}{2N} \approx \frac{W_m}{2N}$$

where p is the frequency of the reference allele at the site X.

Now, consider variable population sizes. Let the haploid population sizes over generations be denoted as $2N_0$, $2N_1$, $2N_2$, … $2N_\infty$, where the subscripts refer to generations back in time, starting just before the sampling generation. The squared covariance D^2 at the sampling generation can be predicted by accumulating the covariances due to mutations from previous generations. The contribution of mutations that occurred in the transition from generation 1 to 0 is:

$$C_0 = D_0^2 = \frac{V_x}{2N_0} \cdot \frac{1}{2N_0} = \frac{W_m}{2N_0}$$

The contribution of mutations that occurred during the transition from generation 2 to generation 1 is:

$$C_1 = \frac{V_x}{4N_1N_1}(1-c)^2\left(1-\frac{2.2}{2N_0}\right) + \frac{V_x}{4N_1N_0}\left(1-\frac{1}{N_0}\right)$$

The first term represents the new covariance from generation 2 to 1, reduced by recombination in the transition from generation 1 to 0 by $(1-c)^2$. The second term is the additional covariance due to sampling remaining variation, i.e., the mutations that occurred from generation 2 to 1, during the transition from generation 1 to 0.

The contribution of mutations that occurred during the transition from generation 3 to 2 is:

$$C_2 = \frac{V_x}{4N_2N_2}\left(1-\frac{2.2}{2N_1}\right)\left(1-\frac{2.2}{2N_0}\right)(1-c)^{2\cdot 2} + \frac{V_x}{4N_2N_1}\left(1-\frac{2.2}{2N_0}\right)(1-c)^2\left(1-\frac{1}{N_1}\right) + \frac{V_x}{4N_2N_0}\left(1-\frac{1}{N_1}\right)\left(1-\frac{1}{N_0}\right)$$

The first term represents the remaining covariance generated by mutations during the transition from generation 3 to 2, the second summand represents the remaining covariance generated during the transition from 2 to 1 and the third represents the remaining covariance generated during the transition from generation 1 to 0.

The general term for the contribution to the observed covariance D^2 in the current generation, due to mutations that occurred during the transition between generation g+1 and g is:

$$C_g = \sum_{i=0}^{g}\left[\frac{V_x}{4N_iN_g}(1-c)^{2i} \cdot \prod_{j=0}^{i-1}\left(1-\frac{2.2}{2N_j}\right) \cdot \prod_{k=i}^{g-1}\left(1-\frac{1}{N_k}\right)\right]$$

where the products of the sequence of terms with negative upper bounds are equal to 1.

The covariance in the present generation, due to all the mutations occurring at sites Y, is the sum of all the C values over all the previous generations, weighted by $\mu \cdot 2N_g$ because the number of mutation events is proportional to the population size:

$$D^2 = \sum_{g=0}^{\infty}(C_g \cdot \mu \cdot 2N_g) = V_x \cdot \mu \cdot \sum_{g=0}^{\infty}\left[\frac{1}{2N_g} \cdot \prod_{i=0}^{g-1}\left[\left(1-\frac{2.2}{2N_i}\right)(1-c)^2\right] \cdot \sum_{j=g}^{\infty}\left[\prod_{i=g}^{j}\left(1-\frac{1}{N_i}\right)\right]\right]$$

where μ is the mutation rate per site.

Similarly, the expected product of variances at sites *X* and *Y* accumulates contributions from all past generations, reduced by drift at a rate N^{-1}:

$$W = \sum_{g=0}^{\infty}\left[\frac{V_x}{2N_g}\cdot\mu\cdot 2N_g\cdot\prod_{i=0}^{g-1}\left(1-\frac{1}{N_i}\right)\right] = V_x\cdot\mu\cdot\sum_{g=0}^{\infty}\left[\prod_{i=0}^{g-1}\left(1-\frac{1}{N_i}\right)\right]$$

Here, $V_x/2N_g$ represents the contribution of mutations at site *Y* to the product of variances W, and $\mu\cdot 2N_g$ is the weight. The product operator represents the decay of the contributions to W over generations.

The expected δ^2 for a given recombination rate *c* is the ratio:

$$\delta_c^2 = \frac{D^2}{W}$$

which becomes independent of both the mutation rate and the standing genic variance. With constant population size, the equation reduces to eq. A4.

1.5- LD in Diploids with Variable Population Size.

In a random mating population of diploids, let *N* be the number of individuals. The contribution of mutations that occurred during the transition from generation 1 to 0 is:

$$C_0 = D_0^2 = \frac{V_x}{2N_0}\cdot\frac{1}{2N_0} = \frac{W_m}{2N_0}$$

The contribution of mutations that occurred during the transition from generation 2 to generation 1 is:

$$C_1 = \frac{V_x}{4N_1N_1}\left(1-\frac{2.2}{2N_0}\right)\cdot[(1-c)^2+c^2] + \frac{V_x}{4N_1N_0}\left(1-\frac{1}{N_0}\right)$$

The first term reflects the new covariance generated between generations 2 and 1, reduced by one round of recombination in the transition from generation 1 to 0 by $(1-c)^2$, and increased by a factor c^2 because genomes are arranged in pairs [see eq. A7, where the terms $D_t'^2$ and W are equal for recent mutations]. The second term accounts for the additional covariance generated by sampling the residual variation, i.e., the mutations that occurred from generation 2 to 1, in the transition from generation 1 to 0.

The contribution of mutations that occurred during the transition from generation 3 to generation 2 is:

$$C_2 = \frac{V_x}{4N_2N_2}\left(1-\frac{2.2}{2N_1}\right)\left(1-\frac{2.2}{2N_0}\right)[(1-c)^2+c^2](1-c)^{2\cdot 1} + \frac{V_x}{4N_2N_1}\left(1-\frac{2.2}{2N_0}\right)[(1-c)^2+c^2]\left(1-\frac{1}{N_1}\right)$$
$$+\frac{V_x}{4N_2N_0}\left(1-\frac{1}{N_1}\right)\left(1-\frac{1}{N_0}\right)$$

The first term is the residual covariance generated by those mutations in the transition from generation 3 to 2, the second summand is the residual covariance generated during

the transition from 2 to 1 and the third summand is the residual covariance generated in the transition from generation 1 to 0.

The contribution of mutations that occur during the transition from generation 4 to generation 3 is:

$$C_3 = \frac{V_x}{4N_3N_3}\left(1-\frac{2.2}{2N_2}\right)\left(1-\frac{2.2}{2N_1}\right)\left(1-\frac{2.2}{2N_0}\right)[(1-c)^2+c^2](1-c)^{2\cdot 2}$$
$$+\frac{V_x}{4N_3N_2}\left(1-\frac{2.2}{2N_1}\right)\left(1-\frac{2.2}{2N_0}\right)[(1-c)^2+c^2](1-c)^{2.1}\left(1-\frac{1}{N_2}\right)$$
$$+\frac{V_x}{4N_3N_1}\left(1-\frac{2.2}{2N_0}\right)[(1-c)^2+c^2]\left(1-\frac{1}{N_2}\right)\left(1-\frac{1}{N_1}\right)+\frac{V_x}{4N_3N_0}\left(1-\frac{1}{N_2}\right)\left(1-\frac{1}{N_1}\right)\left(1-\frac{1}{N_0}\right)$$

The general term for the contribution to the current D^2 of mutations that occurred in the transition between generation g+1 and g, is:

$$C_g \approx \sum_{i=0}^{g}\left[\frac{V_x}{4N_iN_g}(1-c)^{2i}\cdot(1+c^2)\cdot\prod_{j=0}^{i-1}\left(1-\frac{2.2}{2N_j}\right)\cdot\prod_{k=i}^{g-1}\left(1-\frac{1}{N_k}\right)\right]$$

where the products of the sequence of terms with negative upper bounds are equal to 1.

The covariance in the current generation, due to all mutations at sites Y, is the sum of all the C values over all the previous generations, weighted by $\mu\cdot 2N_g$, mutation events are proportional to population size:

$$D^2 \approx \sum_{g=0}^{\infty}(C_g\cdot\mu\cdot 2N_g) = V_x\cdot\mu\cdot(1+c^2)\cdot\sum_{g=0}^{\infty}\left[\frac{1}{2N_g}\cdot\prod_{i=0}^{g-1}\left[\left(1-\frac{2.2}{2N_g}\right)\cdot(1-c)^2\right]\cdot\sum_{j=g}^{\infty}\left[\prod_{i=g}^{j}\left(1-\frac{1}{N_i}\right)\right]\right]$$

The expected value of the product of variances W at sites X and Y is the same as the expectation for a haploid population given in the previous section. The equation for the expected δ^2 for a particular recombination rate c is the ratio of the two contributions, making it independent of both the mutation rate and the standing genetic variance:

$$\delta_c^2 = \frac{D^2}{W}$$

With constant population size, the equation reduces to eq. A10.

1.6- Correction for Sampling Haploids.

We consider the sample as an additional generation of $2n$ haploid individuals (Supplementary Figure 8). Consequently, recombination must be taken into accounted for in the estimation of δ^2.

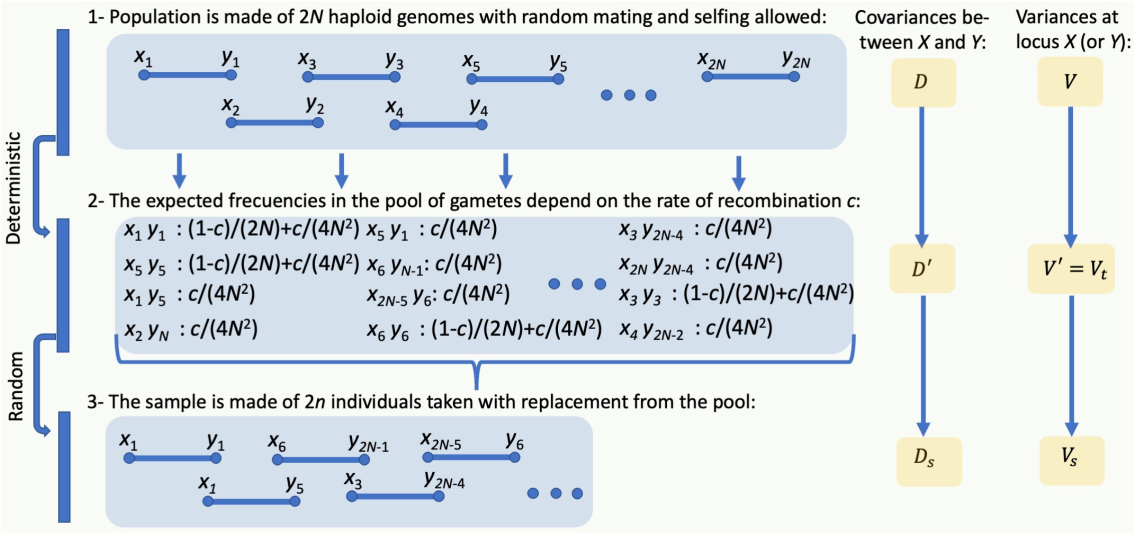

Supplementary Figure 8. Process of sampling of $2n$ haploid individuals in a panmictic population.

Here, we refer to d^2 as the ratio between the means of the observed values D_s^2/W_s in the sample. $\delta^2 = \frac{D}{W}$ is the population parameter to be estimated in the previous generation. Since the sample size $2n$ is typically much smaller than the haploid population size $2N$, we use for sampling the more accurate equations eq. A17 and eq. A19 of this Appendix:

$$E[W_s] \approx W\left(1 - \frac{2}{2n+1}\right)$$

$$E[\,D_s^2] \approx \; D^2\left(1 - \frac{2.2}{2n+1}\right)(1-c)^2 + W\frac{1}{2n+1}$$

Dividing both equations:

$$d_s^2 = \frac{E[\,D_s^2]}{E[W_s]} = \frac{D_t^2\left(1 - \frac{2.2}{2n+1}\right)(1-c)^2 + W\frac{1}{2n+1}}{W\left(1 - \frac{2}{2n+1}\right)}$$

$$= \delta^2\left(1 - \frac{0.2}{2n-1}\right)(1-c)^2 + \frac{1}{2n-1}$$

From this, we derive the estimation of δ^2:

$$\delta^2 = \frac{\left(d_s^2 - \frac{1}{2n-1}\right)}{\left(1 - \frac{0.2}{2n-1}\right)(1-c)^2} \approx \frac{\left(d_s^2 - \frac{1}{2n-1}\right)}{(1-c)^2}$$

1.7- Correction for Sampling Diploids.

While sampling n diploids may appear to be equivalent to sampling $2n$ haploids, this is not entirely true, since in diploids recombination occurs between pairs of fixed haploid genomes. Since the sampling process is equivalent to an additional generation, we follow the rationale outlined in the previous section. From that section, eq. A7 applies because it depends on how haploid genomes are paired in diploid individuals:

$$D'^2 \approx (1-c)^2 D^2 + c^2 \frac{W}{2N}$$

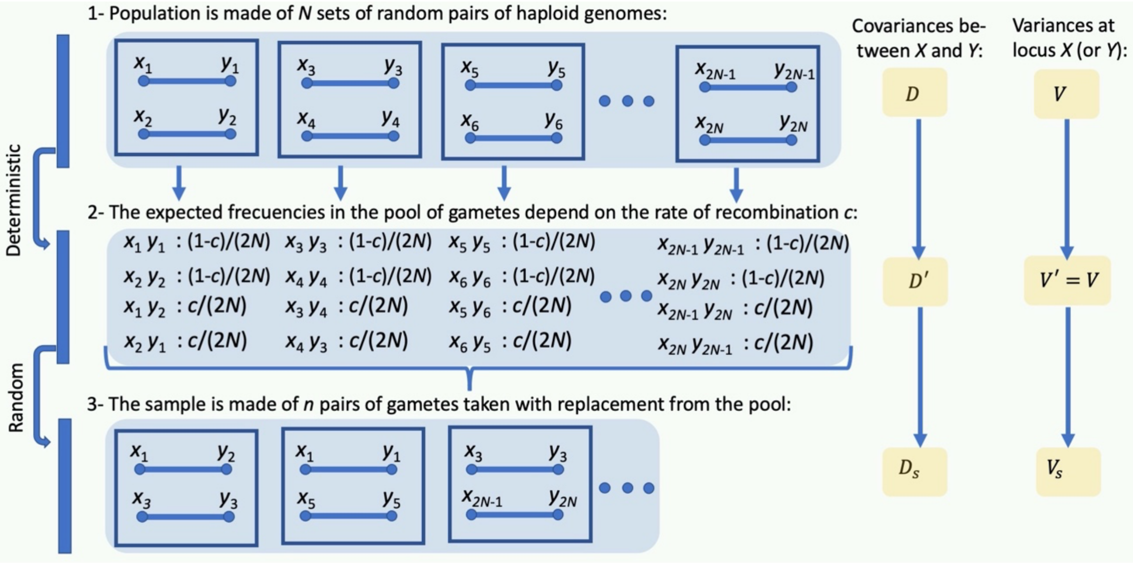

Supplementary Figure 9. Process of sampling of n diploids in a panmictic population when the phase is known..

Equation A8 refers to the random selection of $2N$ gametes to construct the next generation. Here, the sample size n substitutes for the population size N. Since n is usually much smaller than N, we combine eq. A7 with the more precise eq. A19 from the Appendix:

$$E[\,D_s^2] \approx \left[(1-c)^2 D^2 + c^2 \frac{W}{2N}\right]\left(1 - \frac{2.2}{2n+1}\right) + W\frac{1}{2n+1}$$

$$= D^2\left(1 - \frac{2.2}{2n+1}\right)(1-c)^2 + W\left[\frac{1}{2n+1} + \frac{c^2}{2N}\left(1 - \frac{2.2}{2n+1}\right)\right]$$

The expectation of W_s is the same as for haploids:

$$E[W_s] \approx W\left(1 - \frac{2}{2n+1}\right)$$

Dividing both equations we get:

$$d_s^2 = \delta^2\left(1 - \frac{0.2}{2n-1}\right)(1-c)^2 + \frac{1}{2n-1} + \frac{c^2}{2N}$$

Therefore, the estimation for δ^2 *is*:

$$\delta^2 = \frac{\left(d_s^2 - \frac{1}{2n-1} - \frac{c^2}{2N}\right)}{\left(1 - \frac{0.2}{2n-1}\right)(1-c)^2} \approx \frac{\left(d_s^2 - \frac{1}{2n-1}\right)}{(1-c)^2}$$

This approximation is identical to the correction for haploids when *N* is not very small (Section 1.6).

1.8- Correction for Sampling Diploids with Unknown Phase.

When the phase is unknown, it is not possible to calculate the covariance *D* directly from the data. An alternative approach is to calculate the covariance of the bivariate distribution of the means at each locus *X* and *Y* for each of the *n* diploids in the sample:

$$\chi = \frac{x_i + x_j}{2} \quad \text{and} \quad \psi = \frac{y_i + y_j}{2}$$

where subscripts *i* and *j* represent alleles in the two homologous chromosomes of the same individual (see Supplementary Figure 10). The covariance between χ and ψ can be expressed as:

$$cov_{\chi,\omega} = \frac{1}{2} D_s (1 + f)$$

where D_s is the covariance between *x* and *y* values in the haploid genomes of the sample, and *f* is the coefficient of correlation between the allelic values of both haplotypes of the same individual in the sample. The correlation *f* has two components: the intrinsic correlation due to deviations from either random union of gametes or random selection of individuals (f' in Supplementary Figure 10), and the autocorrelation due to the finite size of the sample[4], which is negative ($\approx -1/[2n - 1]$). Thus, a simple estimator of f' would be:

$$\widehat{f'} \approx f + \frac{1}{2n-1}$$

Robertson and Hill[5] found this estimator to be slightly biased. An unbiased estimator of f' is derived in Section 4 of this Appendix:

$$\widehat{f'} = \frac{1 + f(2n-1)}{2n - 1 + f}$$

Burrows' composite measure of LD for pairs of diallelic loci in a sample[6] is given by:

$$\Delta = 2\, P_{AABB} + P_{AABb} + P_{AaBB} + \frac{P_{AaBb}}{2} - 2\, p_A\, p_B$$

Here, capital *P* refers to the frequencies of genotypes in the sample, while lowercase *p* refers to the allelic frequencies at two loci with alleles A/a and B/b, respectively. It can be easily shown that Δ is twice the covariance $cov_{\chi,\omega}$ (with values 1 vs. 0 for the presence vs. absence of a particular allele), which is twice the component of covariance between individuals in the sample ($D_{s_{betw}}$). Here, the samples are diploid instead of haploid. Therefore, replacing 2*n* by *n* in eq. A18 yields:

$$E[\Delta_s^2] = 4 \cdot E\left[D_{s_{betw}}^2\right] \approx 4 \cdot D_{betw}'^2 \cdot \frac{(n-1)^3 + \frac{4}{5}(n-1)^2 + (n-1)}{n^3} + 4 \cdot W_{betw}' \frac{n-1}{n^2}$$

where D'_{betw} is the component of covariance between zygotes in the pool from which the sample was taken. Since $D'_{betw} = {}^1\!/_2\, D'(1 + f')$, where D' is the covariance between the two loci in the pool of gametes from which zygotes are generated, we have:

$$E[\Delta_s^2] \approx D'^2(1+f')^2 \cdot \frac{(n-1)^3 + \frac{4}{5}(n-1)^2 + (n-1)}{n^3} + W'(1+f')^2\frac{n-1}{n^2}$$

The expectation $E[W_s]$ in the sample is (see eq. A16):

$$E[W_s] \approx W'\,\frac{(2n-1)^3 + (2n-1)^2}{8n^3}$$

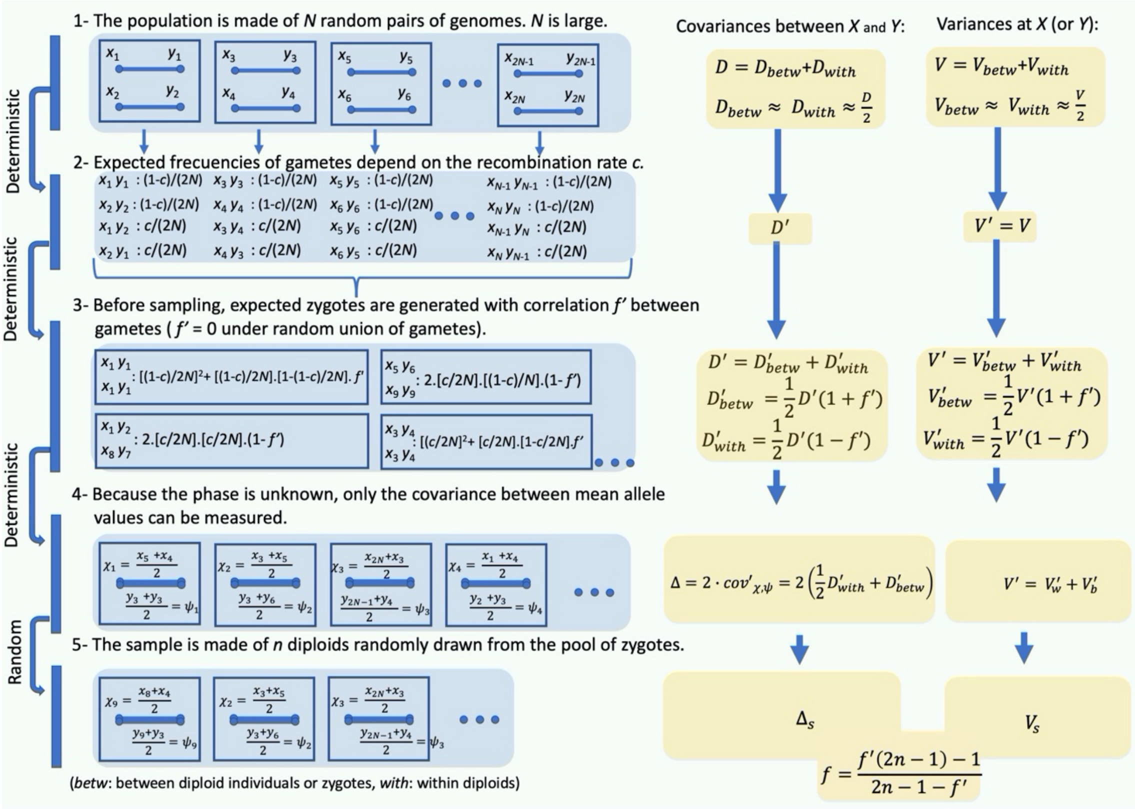

Supplementary Figure 10. Process of sampling in diploid populations when the phase is unknown.

Substituting D'^2 with eq. A7 and dividing the expectations, we get the expectation for d_s^2:

$$d_s^2 = \frac{E[\Delta_s^2]}{E[W_s]} \approx \left[\delta^2(1-c)^2 Z + \frac{c^2}{2N}Z + \frac{4n-4}{(2n-1)^2}\right](1+f')^2$$

where

$$Z = \frac{(2n-2)^3 + \frac{8}{5}(2n-2)^2 + 4(2n-2)}{(2n-1)^3 + (2n-1)^2} \approx \frac{2n-1}{2n}$$

Therefore, the estimation for δ^2 is:

$$\delta^2 = \frac{\frac{d_s^2}{\left(1+f'\right)^2} - \frac{4n-4}{(2n-1)^2} - \frac{c^2}{2N}Z}{Z(1-c)^2} \approx \frac{\frac{d_s^2}{\left(1+f'\right)^2} - \frac{4n-4}{(2n-1)^2}}{Z(1-c)^2}$$

Burrows' composite measure of LD essentially estimates the covariance between loci by considering only the between-individuals component of the covariance. This estimate assumes that the within-individual component has the same value, i.e., $f' = 0$ ($D'_{with} = D'_{betw}$ in Supplementary Figure 10).

1.9- Correction for Low-Coverage Sequencing.

Low coverage can result in a lack of signal from one or both allele copies at a site when sequencing diploid genomes. While the absence of both copies can be detected and corrected by reducing the effective number of individuals in the sample, determining the genotype becomes impossible when only one allelic type is observed. This leads to an increase in observed homozygotes at the expense of heterozygotes. Correcting for this effect in LD estimates is challenging, especially due to coverage differences across individuals and genome regions[7]. However, a simple nonparametric solution based on the pseudo-haploid sampling can be applied by assuming that each of the two copies at heterozygous sites has an equal probability of being identified.

Pseudo-haploid calls are typically obtained by randomly drawing a single allele from each observed heterozygote site[5]. This means that, regardless of whether one or two alleles can be identified, only one allele is considered. This method is equivalent to sampling a single gamete from each diploid individual with a recombination frequency $c = 0.5$ between any pair of loci. Consequently, n pseudohaploid genomes are obtained from n diploid individuals in the sample (Supplementary Figure 11). The derivation is similar to those in previous sections except for the points 4 and 5:

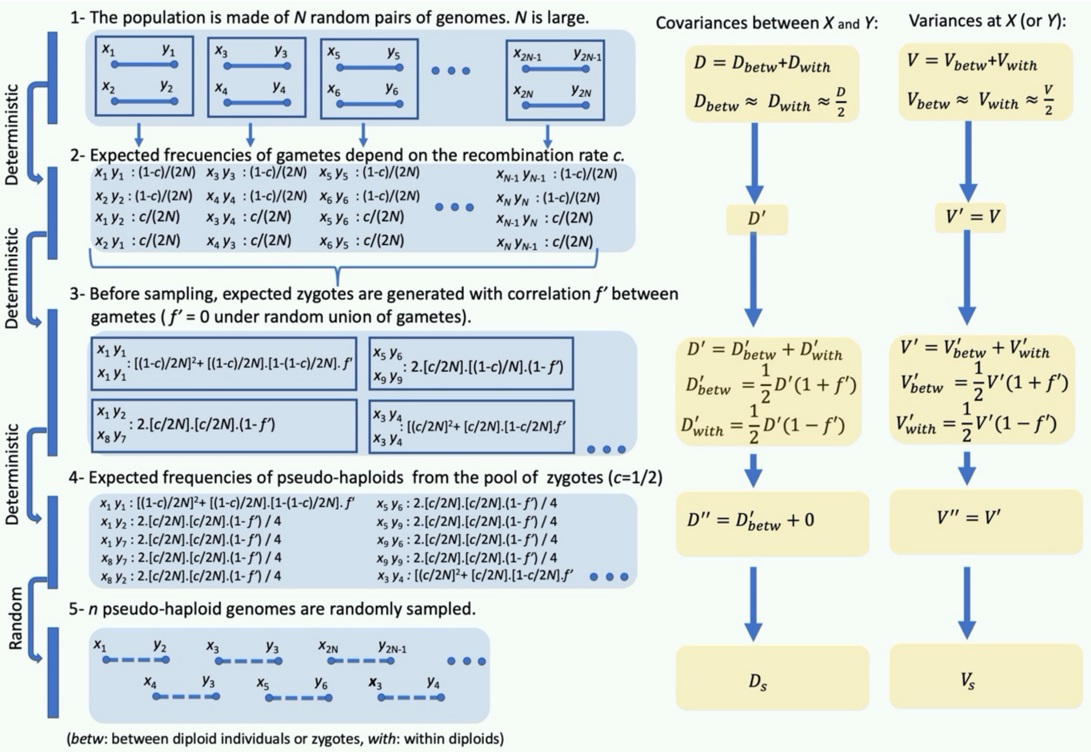

Supplementary Figure 11. Process of sampling of pseudo-haploid genomes.

Point 4 in Supplementary Figure 11 represents the expected frequencies of pseudo-haploid genomes obtained by randomly selecting one of the two alleles during diploid sequencing. The term *f'* is included for completeness although it cannot be estimated from pseudo-haploids data. Applying eq. A19 to the sampling of *n* pseudo-haploids, the expected squared covariance D_s^2 in the sample is:

$$E[D_s^2] = D''^2\left(1 - \frac{2.2}{n+1}\right) + W''\frac{1}{n} = D'^2_{betw}\left(1 - \frac{2.2}{n+1}\right) + W'\frac{1}{n} =$$

$$\frac{1}{4}D'^2\left(1 - \frac{2.2}{n+1}\right) + W'\frac{1}{n}$$

Substituting D'^2 with its value in eq. A7 for diploids:

$$E[D_s^2] = \frac{1}{4}\left[(1-c)^2 D^2 + c^2\frac{W}{2N}\right]\left(1 - \frac{2.2}{n+1}\right) + W\frac{1}{n}$$

The expectation of W_s is the same as for diploids

$$E[W_s] = W\left(1 - \frac{2}{n+1}\right) + D^2\frac{0.2}{n} \approx W\left(1 - \frac{2}{n+1}\right)$$

Then we get the expectation for d_s^2:

$$d_s^2 = \frac{E[D_s^2]}{E[W_s]} = \left(\frac{1}{4}\delta^2(1-c)^2 + \frac{c^2}{8N}\right)\left(1 - \frac{0.2}{n-1}\right) + \frac{1}{n-1}$$

$$d_s^2 \approx \frac{1}{4}\delta^2(1-c)^2\left(1-\frac{0.2}{n-1}\right)+\frac{1}{n-1}$$

Therefore:

$$\delta^2 = 4 \cdot \frac{d_s^2 - \frac{1}{n-1}}{(1-c)^2\left(1-\frac{0.2}{n-1}\right)} \approx 4 \cdot \frac{d_s^2 - \frac{1}{n-1}}{(1-c)^2}$$

GONE2 performs ten rounds of pseudohaploid creation from the entire sample. For each round a δ^2 is estimated for each bin of recombination rate *c* using *n* randomly generated pseudohaploids. The δ^2 values are then averaged over the ten rounds for each bin of *c*. This average is taken as an estimate of the estimate of the δ^2 value in the population.

1.10- Correction for Base-Calling Errors.

Single nucleotide polymorphisms (SNPs) are predominantly di-allelic, so a two-allele model is appropriate to account for genotyping errors caused by allele switching. Let ε be the probability that a call event changes the original allele at a site in an individual, and p the frequency of the reference allele at that site in the population. The expected frequency of the reference allele after sequencing (p') is given by:

$$p' = p + (1-p)\cdot\varepsilon - p\cdot\varepsilon = p + \varepsilon(1-2p)$$

The variance in gene frequency after sequencing (V^*) is expressed as:

$$V^* = [p + \varepsilon(1-2p)] * [(1-p) - \varepsilon(1-2p)] = V[1-4\varepsilon] + \varepsilon$$

From this we can estimate the population variance before sequencing (V):

$$V = \frac{V^* - \varepsilon}{1-4\varepsilon}$$

For two sites *X* and *Y*, the product of variances $W = V_x V_y$ before sequencing is:

$$W = V_x V_y = \frac{V_x^* - \varepsilon}{1-4\varepsilon}\cdot\frac{V_y^* - \varepsilon}{1-4\varepsilon}$$

After introducing errors, W^* becomes:

$$W^* = V_x^* \cdot V_y^*$$

The difference between W and W^* is minimal unless the error rate ε is exceptionally high.

In a di-allelic model, allelic changes during sequencing reduce the observed true population covariance (*D*). An error at either site [with probability $2\varepsilon(1-\varepsilon)$] causes a change in covariance of opposite sign to the true population covariance. However, simultaneous errors at both sites (probability ε^2) do not alter the covariance. Thus, the covariance after sequencing is:

$$D^* = D \cdot [1 - 2\varepsilon(1-\varepsilon)] - D \cdot 2\varepsilon(1-\varepsilon) = D \cdot [1 - 4\varepsilon(1-\varepsilon)]$$

Consequently, the population covariance before sequencing is:

$$D = \frac{D^*}{1 - 4\varepsilon(1-\varepsilon)}$$

And the population value of D^2 before sampling is:

$$D^2 = \frac{{D^*}^2}{[1 - 4\varepsilon(1-\varepsilon)]^2}$$

This equation can be connected to the derivations for sampling haploids (Section 1.6) and sampling diploids (section 1.7), with the result:

$$\delta^2[1 - 4\varepsilon(1-\varepsilon)]^2 \approx \frac{\left(d_s^2 - \frac{1}{2n-1}\right)}{(1-c)^2}$$

for unphased diploids (Section 1.8), with the result:

$$\delta^2[1 - 4\varepsilon(1-\varepsilon)]^2 \approx \frac{\frac{d_s^2}{\left(1+f'\right)^2} - \frac{4n-4}{(2n-1)^2}}{Z(1-c)^2}$$

and for low-coverage (Section 1.9), with the result:

$$\delta^2[1 - 4\varepsilon(1-\varepsilon)]^2 \approx 4 \cdot \frac{d_s^2 - \frac{1}{n-1}}{(1-c)^2}$$

2- Subdivided Populations (Islands Model).

2.1- LD Partition.

Consider a metapopulation composed of *s* subpopulations, each with an identical haploid size 2*N*. Let $x_{i,\alpha}$ and $y_{i,\alpha}$ represent the allele values at sites *X* and *Y*, respectively, in haplotype *i* of subpopulation α. The square of the total covariance of the entire metapopulation at generation *t*, D_t^2, can be expressed in terms of the covariances within subpopulations (D_{w_t}) and the covariance between the means of all subpopulations (D_{b_t}):

$$D_t^2 = \left[\frac{\sum_{\alpha=1}^{s}\sum_{i=1}^{2N}(x_{i,\alpha}-\bar{x})(y_{i,\alpha}-\bar{y})}{s2N}\right]^2$$

$$= \left[\frac{\sum_{\alpha=1}^{s} D_{w\alpha_t}}{s} + \frac{\sum_{\alpha=1}^{s}(\bar{x}_\alpha-\bar{x})(\bar{y}_\alpha-\bar{y})}{s}\right]^2 = \left[D_{w_t} + D_{b_t}\right]^2$$

where $\bar{x}_\alpha = \frac{\sum_{i=1}^{2N} x_{i,\alpha}}{2N}$, $\bar{y}_\alpha = \frac{\sum_{i=1}^{2N} y_{i,\alpha}}{2N}$, $\bar{x} = \frac{\sum_{\alpha=1}^{s} \bar{x}_\alpha}{s}$, and $\bar{y} = \frac{\sum_{\alpha=1}^{s} \bar{y}_\alpha}{s}$. Note that D_{w_t} is the average of the *s* covariances within subpopulations, relative to their respective means for the variables *X* and *Y*. In terms of sampling and drift, D_{w_t} is equivalent to the covariance of a single population *s* times the size of each subpopulation, except for a small fraction of the order 1/*N* due to autocorrelation. Expanding the equation, we have:

$$D_t^2 = D_{w_t}^2 + 2 \cdot D_{bw_t}^2 + D_{b_t}^2 \tag{A11}$$

where $D_{bw_t}^2 = \left[D_{w_t} \cdot D_{b_t}\right]$ is the expectation of the product of the within and between covariances.

Similarly, the product of the genic variances W_t=$V_{x_t}V_{y_t}$ at two sites *X* and *Y* in the entire metapopulation can be decomposed into components between (*b*) and within (*w*) subpopulations:

$$W_t = \frac{\sum_{\alpha=1}^{s}\sum_{i=1}^{2N}(x_{i,\alpha}-\bar{x})^2}{s2N} \cdot \frac{\sum_{\alpha=1}^{s}\sum_{i=1}^{2N}(y_{i,\alpha}-\bar{y})^2}{s2N}$$

$$= \left[\frac{\sum_{\alpha=1}^{s} V_{x\alpha}}{s} \cdot \frac{\sum_{\alpha=1}^{s} V_{y\alpha}}{s}\right] + \left[\frac{\sum_{\alpha=1}^{s} V_{x\alpha}}{s} \cdot \frac{\sum_{\alpha=1}^{s}(\bar{y}_\alpha-\bar{y})^2}{s} + \frac{\sum_{\alpha=1}^{s} V_{y\alpha}}{s} \cdot \frac{\sum_{\alpha=1}^{s}(\bar{x}_\alpha-\bar{x})^2}{s}\right]$$

$$+ \left[\frac{\sum_{\alpha=1}^{s}(\bar{x}_\alpha-\bar{x})^2}{s} \cdot \frac{\sum_{\alpha=1}^{s}(\bar{y}_\alpha-\bar{y})^2}{s}\right]$$

$$= [W_w] + \left[V_{x,w_t}V_{y,b_t} + V_{y,w_t}V_{x,b_t}\right] + \left[W_{b_t}\right]$$

2.2- Expectations of LD Components for Haploids and Diploids.

Let us assume that the metapopulation is at mutation-migration-drift equilibrium, so the squared covariances $D^2_{w\,t}$, $D^2_{bw\,t}$, and $D^2_{b\,t}$, and the product of the variances W_t remain constant over generations. Let m be the symmetrical migration rate between subpopulations. The evolution of the parameters is a superposition of deterministic and random changes (Supplementary Figure 12). Deterministic changes after one round of recombination and migration are:

$$D'_{w\,t} = D_{w\,t}(1-c) + \left(\frac{s}{s-1}\right)^2 \cdot D_{b\,t} \cdot m(1-m)$$

$$D'_{b\,t} = D_{b\,t} \cdot \left(1 - \frac{s}{s-1} m\right)^2$$

Note that recombination occurs before migration, i.e. it does not reduce the component of covariance between subpopulations.

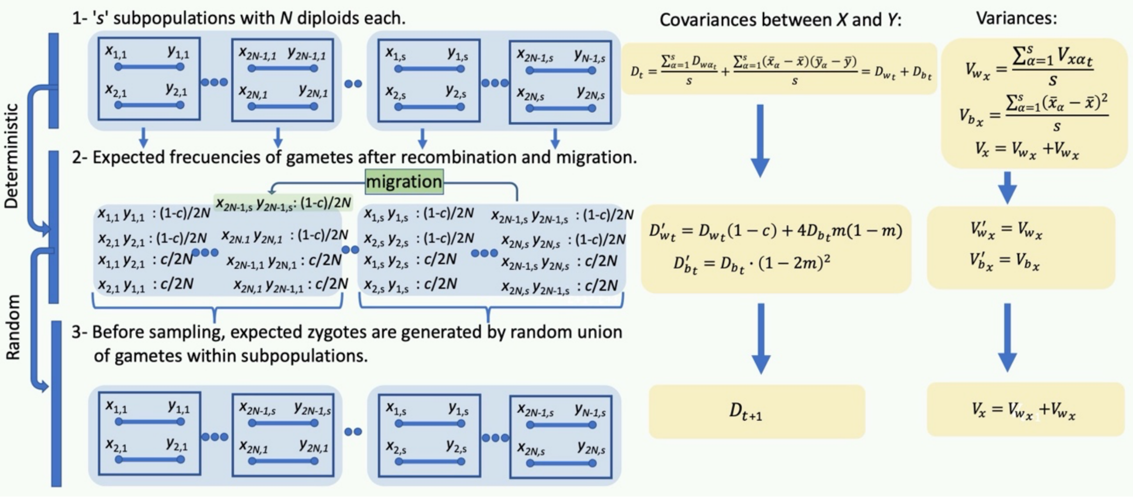

Supplementary Figure 12. Process of transition from one generation to the next in a subdivided population of diploid individuals. Variances at both sites are equivalent and are considered constant.

The corresponding squared covariances after the deterministic change but before sampling are:

$$D'^2_{w\,t} = D^2_{w\,t} \cdot (1-c)^2 + \left(\frac{s}{s-1}\right)^4 \cdot D^2_{b\,t} \cdot m^2(1-m)^2 + 2\left(\frac{s}{s-1}\right)^2 \cdot D^2_{bw\,t} \cdot m(1-m)(1-c)$$

$$D'^2_{b\,t} = D^2_{b\,t} \cdot \left(1 - \frac{s}{s-1} m\right)^4$$

$$D'^2_{bw\,t} = D'_{b\,t} \cdot D'_{w\,t} = D^2_{bw\,t} \cdot \left(1 - \frac{s}{s-1} m\right)^2 (1-c) + \left(\frac{s}{s-1}\right)^2 \cdot D^2_{b\,t} \cdot m(1-m)\left(1 - \frac{s}{s-1} m\right)^2$$

Subsequently, the squared covariance within subpopulations is expected to change due to the sampling of the $s2N$ haploids that make up the next generation (eq. A19 refers here to the entire metapopulation):

$$E\left[D^2_{w\,t+1}\right] = D'^2_{w\,t} \cdot \left(1 - \frac{2.2}{s2N}\right) + W_w \cdot \frac{1}{s2N}$$

For diploids, this equation takes the form (see derivation of eq. A8, which here refers to the entire metapopulation):

$$E\left[D^2_{w_{t+1}}\right] = D'^2_{w\,t} \cdot \left(1 - \frac{2.2}{s2N}\right) + W_w \cdot \left(\frac{1}{s2N} + \frac{c^2}{s2N}\right)$$

Also,

$$E\left[D^2_{bw_{t+1}}\right] = D'^2_{bw_t} \cdot \left(1 - \frac{1}{sN}\right) + \frac{D^2_{w_t}}{s2N}$$

At equilibrium, the value for $\widehat{D}^2_b$ can be approximated as follows:

$$\widehat{D}^2_b = \left(\frac{\sum_{\alpha=1}^{s}(\bar{x}_\alpha - \bar{x}) \cdot (\bar{y}_\alpha - \bar{y})}{s}\right)^2 = \frac{\left[\sum_{\alpha=1}^{s}(\bar{x}_\alpha - \bar{x})^2 \cdot (\bar{y}_\alpha - \bar{y})^2\right]/s}{s-1} = \frac{E[(\bar{x}_\alpha - \bar{x})^2]^2}{s-1} + \epsilon^2$$
$$= \frac{E[(\bar{y}_\alpha - \bar{y})^2]^2}{s-1} + \epsilon^2 \approx \frac{\hat{F}^2_{ST}}{s-1} \cdot \widehat{W}$$

The approximation assumes that the variation between subpopulations is uncorrelated between sites, which is fundamentally true if the sites are not tightly linked, and the deviation ϵ^2 is small. Also, under this assumption, $\widehat{W}_w \approx \widehat{W} \cdot \left(1 - \hat{F}_{ST}\right)^2$, which simplifies the derivation and leads to interpretable equations while retaining relevant terms for precision.

Arranging the above equations at equilibrium, i.e., making $\widehat{D}^2_w = D^2_{w_t} = D^2_{w_{t+1}}$ and $\widehat{D}^2_{bw} = D^2_{bw_t} = D^2_{bw_{t+1}}$, we finally have for *s* subpopulations of 2*N* haploids:

$$\widehat{D}^2_w = \widehat{W} \cdot \left(1 - \hat{F}_{ST}\right)^2 \cdot \frac{1}{s2N\,(1 - (1-c)^2 + 2.2\,(1-c)^2}$$

$$\widehat{D}^2_{bw} = \frac{\left(\frac{s}{s-1}\right)^2 \widehat{D}^2_b \cdot m(1-m)\left(1 - \frac{s}{s-1}m\right)^2 + \widehat{D}^2_w/_{s2N}}{1 - \left(1 - \frac{s}{s-1}m\right)^2 \cdot (1-c) \cdot \left(1 - 1/_{sN}\right)}$$
$$\approx \frac{\left(\frac{s}{s-1}\right)^2 \widehat{D}^2_b \cdot m}{1 - \left(1 - \frac{s}{s-1}m\right)^2 \cdot (1-c)}$$

$$\widehat{D}^2_b = \widehat{W} \cdot \frac{\hat{F}^2_{ST}}{s-1}$$

$$\widehat{D}^2 = \widehat{D}^2_w + 2 \cdot \widehat{D}^2_{bw} + \widehat{D}^2_b$$

Dividing both sides of the latter equation by $\widehat{W}$, we get the following for haploids:

$$\hat{\delta}^2 = \hat{\delta}^2_w + 2 \cdot \hat{\delta}^2_{bw} + \hat{\delta}^2_b =$$
$$\left(1 - \hat{F}_{ST}\right)^2 \cdot \frac{1}{s2N\,(1 - (1-c)^2 + 2.2\,(1-c)^2} + 2 \cdot \frac{\left(\frac{s}{s-1}\right)^2 \hat{F}^2_{ST} \cdot m}{1 - \left(1 - \frac{s}{s-1}m\right)^2 (1-c)} + \frac{\hat{F}^2_{ST}}{s-1}$$

(A12)

The equivalent equation for diploids is:

$$\hat{\delta}^2 = \left(1-\hat{F}_{ST}\right)^2 \cdot \frac{1+c^2}{s2N\left(1-(1-c)^2\right)+2.2\,(1-c)^2} + 2\cdot\frac{\left(\frac{s}{s-1}\right)^2 \hat{F}_{ST}^2 \cdot m}{1-\left(1-\frac{s}{s-1}m\right)^2(1-c)} + \frac{\hat{F}_{ST}^2}{s-1}$$

(A13)

If the recombination rate is not small, $\hat{\delta}_{bw}^2$ is negligible and can be ignored. Table S1, in the Supplementary Tables, shows simulation results and predictions using these equations for various subpopulation sizes, migration rates, and recombination rates. Assuming equilibrium, $\hat{F}_{ST}$ can be predicted using the equation of Takahata[9]:

$$\hat{F}_{ST} = \frac{1}{4N\cdot m\cdot\left(\frac{s}{s-1}\right)^2+1}$$

(A14)

2.3- Correction for Sampling Diploids and Haploids.

The model assumes that both subpopulations are of equal size, with random sampling likely lead to overrepresentation of one subpopulation in the sample. This assumption allows similar sampling corrections to be applied as in panmictic populations, with the key difference that recombination only affects the within-subpopulations component of the covariance D'_w (as shown in Section 2.2, Figure A7).

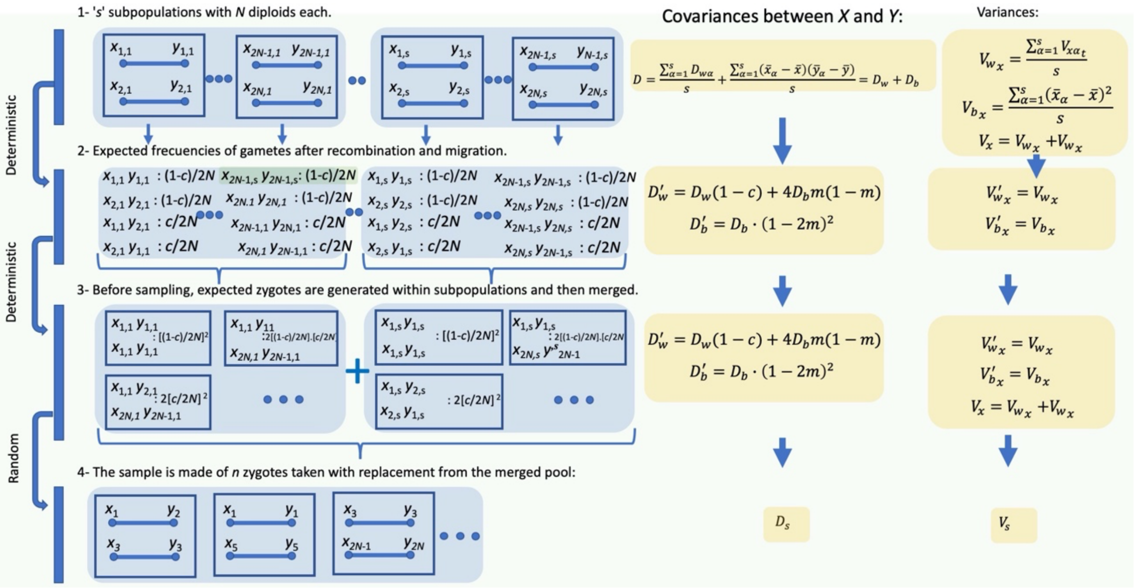

Supplementary Figure 13. Process of sampling of diploids in a metapopulation.

Supplementary Figure 13 illustrates the process of sampling diploids in a metapopulation when the phase is known. The figure shows how recombination and migration operate prior sampling, changing the expectations of the covariances D'_w and D'_b (points 1, 2 and 3). The squared covariances after deterministic changes due to recombination and migration are:

$$D_w'^2 = D_w^2\cdot(1-c)^2 + \left(\frac{s}{s-1}\right)^4\cdot D_b^2\cdot m^2(1-m)^2 + 2\left(\frac{s}{s-1}\right)^2\cdot D_{bw}^2\cdot m(1-m)(1-c)$$

$$D_b'^2 = D_b^2 \cdot (1 - \frac{s}{s-1} m)^4$$

$$D_{bw}'^2 = D_b' \cdot D_w' = D_{bw}^2 \cdot \left(1 - \frac{s}{s-1} m\right)^2 (1-c) + \left(\frac{s}{s-1}\right)^2 \cdot D_b^2 \cdot m(1-m) \left(1 - \frac{s}{s-1} m\right)^2$$

The total squared covariance before sampling is:

$$D'^2 = D_w'^2 + 2 \cdot D_{bw}'^2 + D_b'^2$$

Dividing both sides of the equation by W gives the total LD before sampling:

$$\delta'^2 = \delta_w'^2 + 2 \cdot \delta_{bw}'^2 + \delta_b'^2$$

The correction for sampling is similar to that for a panmictic diploid population (Section 1.7), except for the recombination term, which is already included in D_w' and is shown in point 2 of Figure 8. Therefore:

$$\delta^2 = d_s^2 - \frac{1}{2n-1}$$

where $d_s^2 = \frac{D_s^2}{W_s}$ is the observed LD in the sample, and $\delta^2 = \frac{D}{W}$ is the LD to be estimated. This correction applies for both haploids and phased diploids.

2.4- Correction for Sampling Diploids with Unknown Phase.

When the phase is unknown, only the covariance of the bivariate distribution of the means at each locus (χ and ψ) can be calculated (see Section 1.8). The first three points of the sampling process shown in Supplementary Figure 14 correspond to the first three points of the panmictic populations in Figure 5, with the addition of migration affecting the expectations of the covariances.

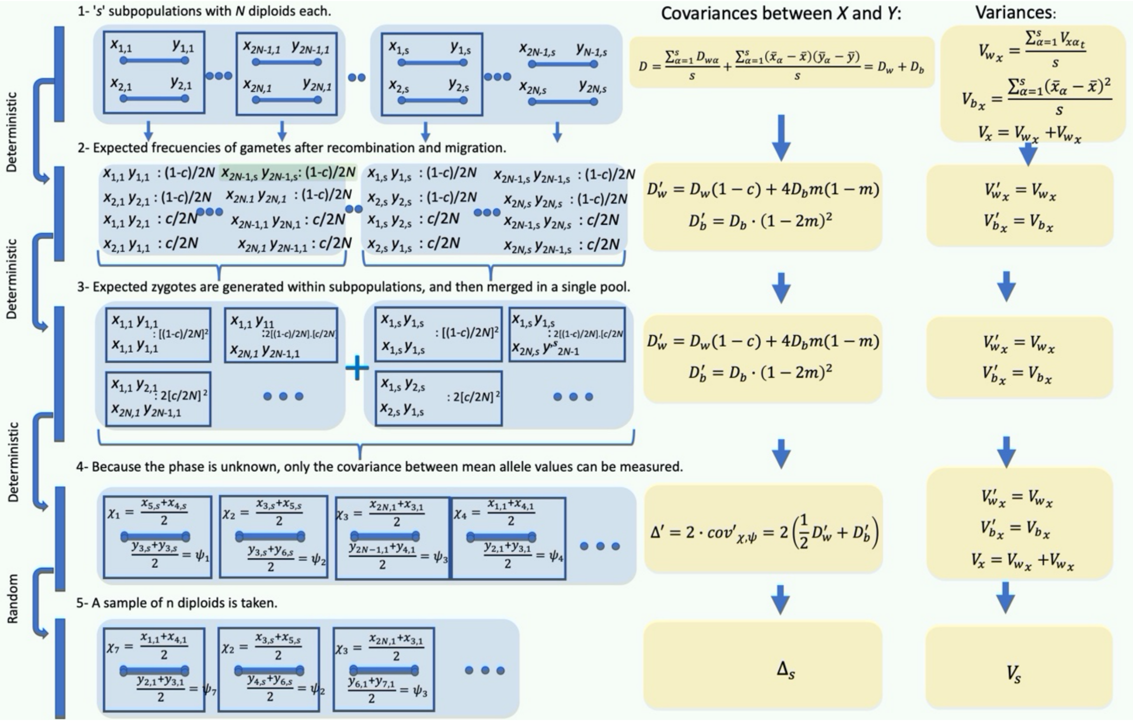

Supplementary Figure 14. Process of sampling of unphased diploids in a metapopulation.

After merging the two subpopulations before sampling (point 4 in Supplementary Figure 14), the expected covariance of the means is:

$$cov'_{\chi,\omega} = \frac{1}{2}D'_w + D'_b$$

The product of variances between individuals before sampling W'_{betw} is:

$$W'_{betw} = \left(\frac{1}{2}V'_w + V'_b\right)^2 = W' \cdot (1+f')^2$$

The square of Burrows' composite measure of LD before sampling is:

$$\Delta'^2 = \left(2 \cdot cov'_{\chi,\omega}\right)^2 = D'^2_w + 4 \cdot D'^2_{bw} + 4 \cdot D'^2_b$$

After sampling (see Section 1.8):

$$E[\Delta_s^2] \approx \Delta'^2 \cdot \frac{(n-1)^3 + \frac{4}{5}(n-1)^2 + (n-1)}{n^3} + 4 \cdot W'_{betw}\frac{n-1}{n^2}$$

and,

$$E[W_s] \approx W' \frac{(2n-1)^3 + (2n-1)^2}{8n^3}$$

The ratio of these two expressions is the LD expected in the sample:

$$d_s^2 = \frac{E[\Delta_s^2]}{E[W_s]} = \delta^2 \cdot Z + \frac{4n-4}{(2n-1)^2} \cdot (1+f')^2$$

2.5- Correction for Pseudo-haploids.

In Section 1.9 (Correction for Depth Coverage), the haploidization of the sample by randomly selecting one allele in heterozygotes is equivalent to an additional round of recombination at a rate of 0.5, regardless of the distance between marker sites. This pseudorecombination event within diploid genomes of the same subpopulation halves the expectation of the within-subpopulation covariance D''_w (point 4 in Supplementary Figure 15) but does not affect the between-subpopulation component D''_b.

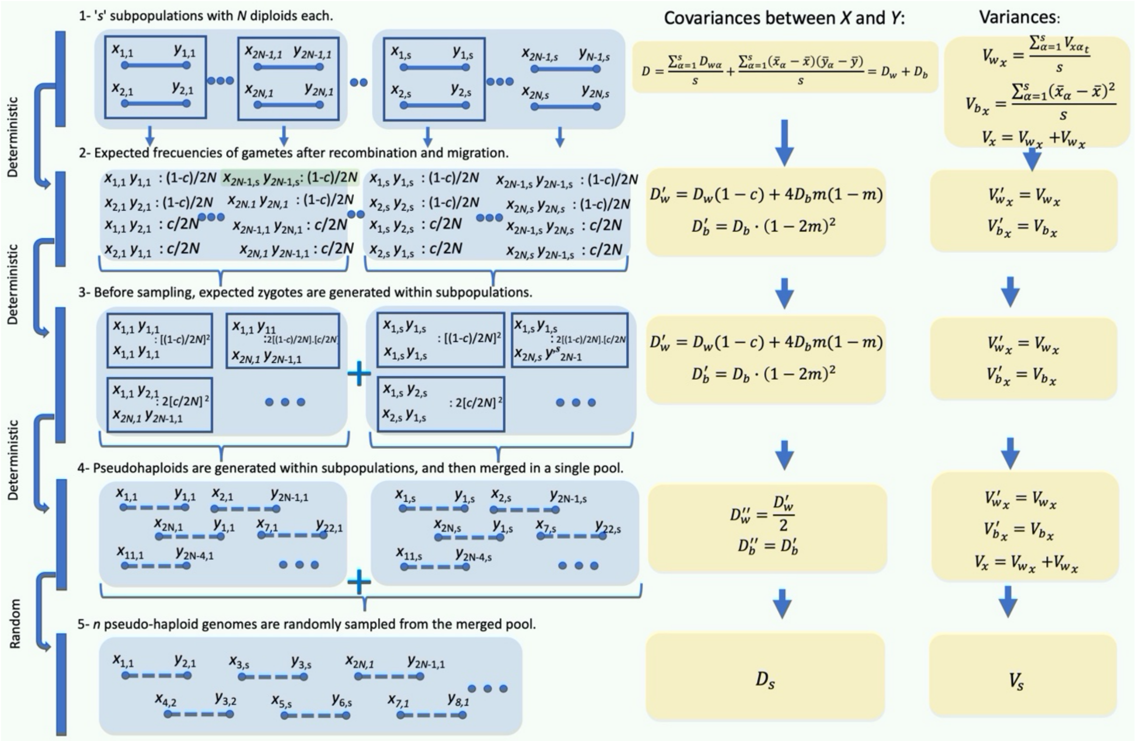

Supplementary Figure 15. Process of sampling of pseudo-haploid genomes in a metapopulation.

Consequently, the pre-sampling expectation of D''^2_{bw} is also halved, and the expectation of D''^2_w is reduced to a quarter: Thus, when sampling pseudo-haploid genomes, the squared covariance before sampling is reduced to:

$$D''^2 = D'^2_w \cdot \frac{1}{4} + 2 \cdot D'^2_{bw} \cdot \frac{1}{2} + D'^2_b$$

Dividing by W, we get:

$$\delta''^2 = \delta'^2_w \cdot \frac{1}{4} + 2 \cdot \delta'^2_{bw} \cdot \frac{1}{2} + \delta'^2_b$$

The sampling correction is applied here in the same way as described in Section 1.9 "Correction for Low Coverage Sequencing", but without the terms for recombination and haploidization, which are already included in the above derivation:

$$\delta''^2 = d_s^2 - \frac{1}{n-1}$$

where $d_s^2 = \frac{D_s^2}{W_s}$ is the LD observed in the sample.

3- Some Statistical Equations.

Consider a bivariate distribution (x,y) in an infinite population with joint central moments of order (r, s):

$$\mu_{rs} = E\left[(x-\mu_x)^r\left(y-\mu_y\right)^s\right] = E[x'^r y'^s]$$

This includes the marginal moments:

$$\mu_{r0} = E[(x-\mu_x)^r] = E[x'^r] \text{ and } \mu_{0s} = E\left[\left(y-\mu_y\right)^s\right] = E[y'^s]$$

A set of $2n$ bivariate values (x_1, y_1), (x_2, y_2), … (x_{2n}, y_{2n}) is randomly taken from this distribution, where $2n$ represents either the population size or the sample size, as appropriate for the context. From these values, the following expectations can be derived (see the Supplementary File in Santiago et al. (2020)[1], for a detailed derivation):

3.1- Expectation of the Sample Product of Two Variances.

Let W be the product of the two sample variances,

$$W = V_x \cdot V_y = \frac{\sum_i (x_i-\bar{x})^2}{2n} \cdot \frac{\sum_i (y_i-\bar{y})^2}{2n}$$

where $\bar{x} = \frac{\sum_i x_i}{2n}$ and $\bar{y} = \frac{\sum_i y_i}{2n}$.

It can be shown that the expectation of the product of the variances in the sample is

$$E[W] = \mu_{20}\mu_{02}\frac{(2n-1)^3}{8n^3} + \mu_{22}\frac{(2n-1)^2}{8n^3} + \mu_{11}^2\frac{2n-1}{4n^3}$$

Ignoring terms of order n^{-2}:

$$E[W] \approx \mu_{20}\mu_{02}\frac{(2n-1)^3}{8n^3} + \mu_{22}\frac{(2n-1)^2}{8n^3} \quad \text{(A15)}$$

Substituting μ_{22} using eq. A21, and considering $\frac{\mu_{11}^2}{2n} \ll \mu_{20}\mu_{02}$:

$$E[W] = \mu_{20}\mu_{02}\frac{(2n-1)^3 + (2n-1)^2}{8n^3} + \frac{4}{5}\frac{(2n-1)^2}{8n^3}\mu_{11}^2 \approx \mu_{20}\mu_{02}\frac{(2n-1)^3 + (2n-1)^2}{8n^3} \quad \text{(A16)}$$

And if n is not small:

$$E[W] \approx \mu_{20}\mu_{02}\left(1 - \frac{2}{2n+1}\right) \approx \mu_{20}\mu_{02}\left(1 - \frac{1}{n}\right) \quad \text{(A17)}$$

3.2- Expectation of the Square of the Sample Covariance.

Let D be the sample covariance

$$D = \frac{\sum_i (x_i-\bar{x})(y_i-\bar{y})}{2n}$$

The expectation of D^2 is:

$$E[D^2] = \mu_{11}^2\frac{(2n-1)^3 + (2n-1)}{8n^3} + \mu_{22}\frac{(2n-1)^2}{8n^3} + \mu_{20}\mu_{02}\frac{2n-1}{8n^3}$$

Substituting μ_{22} using eq. A21:

$$E[D^2] \approx \mu_{11}^2 \frac{(2n-1)^3 + \frac{4}{5}(2n-1)^2 + (2n-1)}{8n^3} + \mu_{20}\mu_{02}\frac{2n-1}{4n^2}$$

(A18)

And if n is not small:

$$E[D^2] \approx \mu_{11}^2\left(1 - \frac{2.2}{2n+1}\right) + \mu_{20}\mu_{02}\frac{1}{2n+1} \approx \mu_{11}^2\left(1 - \frac{2.2}{2n}\right) + \mu_{20}\mu_{02}\frac{1}{2n}$$

(A19)

3.3- Expectation for the Sample Moment m_{22}.

Let m_{22} be the (2,2)th central moment of the sample:

$$E[m_{22}] = E\left[\frac{\sum_i[(x_i - \bar{x})^2(y_i - \bar{y})^2]}{2n}\right]$$

The expansion of the expectation leads to:

$$E[m_{22}] = \mu_{22}\left[1 - \frac{2}{n} + \frac{3}{2n^2} - \frac{3}{8n^3}\right] + \mu_{20}\mu_{02}\left[\frac{2n-1}{2n^2} - 3\frac{2n-1}{8n^3}\right] + \mu_{11}^2\left[\frac{2n-1}{n^2} - 6\frac{2n-1}{8n^3}\right]$$

Ignoring terms of order n^{-2}:

$$E[m_{22}] \approx \mu_{22}\left[1 - \frac{2}{n}\right] + \mu_{20}\mu_{02}\left[\frac{2n-1}{2n^2}\right] + \mu_{11}^2\left[\frac{2n-1}{n^2}\right]$$

(A20)

3.4- Approximation to the Population Moment μ_{22} at Equilibrium.

We want to approximate the (2,2)th central moment of a population with size $2N$ in a continuous process where offspring replace parents. In this dynamical system, the new variation introduced by mutation is uncorrelated between the two variables. The process eventually reaches an equilibrium state where all moments remain invariant across generations. When the two variables are uncorrelated, we have:

$$\mu_{22} = \mu_{20}\mu_{02} \quad \text{and} \quad m_{22} = W\ ,$$

However, if they are correlated:

$$\mu_{22} = \mu_{20}\mu_{02} + \chi \quad \text{and} \quad m_{22} = W + X$$

where χ and X represent deviations.

Therefore, eq. A20 can be rewritten:

$$E[m_{22}] \approx E[W + X\ ] = (\mu_{20}\mu_{02} + \chi)\left[1 - \frac{2}{N}\right] + \mu_{20}\mu_{02}\left[\frac{2N-1}{2N^2}\right] + \mu_{11}^2\left[\frac{2N-1}{2N^2}\right]$$

Substituting W using eq. A15:

$$\mu_{20}\mu_{02}\frac{(2N-1)^3}{8N^3} + (\mu_{20}\mu_{02} + \chi)\frac{(2N-1)^2}{8N^3} + E[X]$$
$$\approx (\mu_{20}\mu_{02} + \chi)\left[1 - \frac{2}{N}\right] + \mu_{20}\mu_{02}\left[\frac{1}{N}\right] + \mu_{11}^2\left[\frac{2N-1}{N^2}\right]$$

Simplifying, we find:

$$\mu_{20}\mu_{02}\left(1 - \frac{1}{N}\right) + \chi\frac{1}{2N} + E[X] \approx \mu_{20}\mu_{02}\left[1 - \frac{1}{N}\right] + \chi\left[1 - \frac{2}{N}\right] + \mu_{11}^2\left[\frac{2}{N}\right]$$

Thus:

$$E[X] \approx \chi\left[1-\frac{5}{2N}\right]+\mu_{11}^{2}\left[\frac{2}{N}\right]$$

Unless the population size $2N$ is very small, new variation introduced in any generation represents only a small fraction of the total genetic variation. Consequently, most of the disequilibrium in the population builds on ancestral variation. This characteristic implies that the difference ε between χ (after introducing variation) and. X (before) is very small:

$$E[X] \approx \chi-\varepsilon \approx \chi \approx \chi\left[1-\frac{5}{2N}\right]+\mu_{11}^{2}\left[\frac{2}{N}\right]$$

Therefore:

$$\chi \approx \frac{4}{5}\mu_{11}^{2}$$

Thus, the approximation for μ_{22} is:

$$\mu_{22} \approx \mu_{20}\mu_{02}+\frac{4}{5}\mu_{11}^{2} \tag{A21}$$

4- Estimation of the Inbreeding Coefficient.

We define the inbreeding coefficient of either a population or a metapopulation in terms of deviations from Hardy-Weinberg expectations:

$$f'=\frac{P-p^{2}}{p(1-p)}$$

where p is the frequency of a particular allele and P is the frequency of homozygotes for that allele. This definition is the same as the correlation F_{IT} between gametes within individuals relative to the entire metapopulation.

Haldane[4] derived an equation to estimate the inbreeding coefficient f' of a monoecious population from the observed coefficient f in a sample of n diploids. After some simplifications his equation reduces to:

$$E[f']=f+\frac{1}{2n-1}$$

Roberson and Hill[5] pointed out that the equation is biased by an amount of the order of $f'/2n$. Obviously, the bias can be ignored if n is not small but, for some purposes, more accurate estimates are needed. What follows is the derivation of the unbiased estimator of f'.

First we consider an infinitely large population of diploid individuals and a diallelic locus with the definition:

$$f'=\frac{\overline{[P-p^{2}]}}{\overline{[p(1-p)]}}$$

where the averages can refer either to the true values of one locus or to the averages over several loci. In fact $[P-p^{2}]$ is not really a covariance between two variables (say,

maternal and paternal gametes), but here the difference is zero because the population is infinite. Another problem is that the equation is a ratio of expectations rather than an expectation of a ratio, which is not a problem for a particular locus but could make a slight difference if the derivation is extended to a set of different loci.

Now we consider the sample of *n* diploids for which we define:

$$f = \frac{\overline{[X - x^2]}}{\overline{[x(1-x)]}}$$

where *x* is the frequency of the reference allele and *X* is the frequency of homozygotes for the same allele in the sample. Here, the averages are calculated over multiple loci. We have verified (data not shown) that as few as ten loci are sufficient to obtain virtually unbiased estimates of the "true" *f* value in the sample when loci are randomly sampled from populations in mutation-drift equilibrium.

As we have pointed out, $E[X - x^2]$ is not really a covariance, and this fact becomes more obvious as the sample becomes smaller. We can express:

$$E[X - x^2] = E[X - (\bar{x}_1 + \Lambda) \cdot (\bar{x}_2 - \Lambda)] = E[X - \bar{x}_1\bar{x}_2] - E[\Lambda^2]$$

where the subscripts 1 and 2 represent the series of maternal and paternal gametes respectively and $\Lambda = (\bar{x}_2 - \bar{x}_1)/2$. The term $E[X - \bar{x}_1\bar{x}_2]$ is the covariance between the two variables 1 and 2 in the sample and, therefore, we can write:

$$E[X - \bar{x}_1 \cdot \bar{x}_2] = E[P - p^2]\frac{n-1}{n}$$

and

$$E[\Lambda^2] = E\left[\frac{\bar{x}_2 - \bar{x}_1}{2}\right] = \frac{E[V_{smp\_with}]}{n} = \frac{\overline{V_{pop\_with}}}{n} = \frac{\frac{\overline{V_{pop}}(1-f')}{2}}{n} = \frac{\overline{[p(1-p)]}(1-f')}{2n}$$

where V_{smp_with}, V_{pop_with} and V_{pop} are respectively the component of variation within individuals in the sample, the component of variation within individuals in the population, and the total variation in the population, respectively.

Also $E[x(1-x)]$ can be given in terms of $[p(1-p)]$:

$$\begin{aligned} E[x(1-x)] &= V_{smp\_with} + V_{smp\_betw} = V_{pop\_with} + V_{pop\_betw}\frac{n-1}{n} \\ &= \frac{1}{2}\overline{[p(1-p)]}(1-f') + \frac{1}{2}\overline{[p(1-p)]}(1+f')\frac{n-1}{n} \\ &= \frac{1}{2}\overline{[p(1-p)]} \cdot \left[(1-f') + (1+f')\frac{n-1}{n}\right] \end{aligned}$$

where V_{smp_betw} and V_{pop_betw} are the components of variance between individuals in the sample and in the population respectively. Finally, we get:

$$f = \frac{E[X - x^2]}{E[x(1-x)]} = \frac{\overline{[P - p^2]} \cdot \frac{n-1}{n} - \frac{\overline{[p(1-p)]}(1-f')}{2n}}{\frac{1}{2}\overline{[p(1-p)]} \cdot \left[(1-f') + (1+f')\frac{n-1}{n}\right]}$$

$$f = \frac{f' \cdot \frac{n-1}{n} - \frac{(1-f')}{n}}{\frac{1}{2} \cdot \left[(1-f') + (1+f')\frac{n-1}{n}\right]} = \frac{f'(2n-1) - 1}{2n - 1 - f'}$$

(A22)

Therefore, the estimator of the coefficient in the population is:

$$\widehat{f'} = \frac{1 + f(2n-1)}{2n - 1 + f}$$

(A23)

5- Supplementary References.

1. Santiago, E., Novo, I., Pardiñas, A.F., Saura, M., Wang, J. & Caballero, A. Recent Demographic History Inferred by High-Resolution Analysis of Linkage Disequilibrium. *Mol. Biol. Evol.* **37**, 3642-3653 (2020).
2. Ohta T. & Kimura M. Linkage disequilibrium due to random genetic drift. *Genet. Res.* **13**, 47-55 (1969).
3. Santiago, E., Caballero, A., Köpke, C. & Novo, I. Estimation of the contemporary effective population size from SNP data while accounting for mating structure. *Mol. Ecol. Resour.* **24**, e13890 (2024).
4. Haldane, J.B.S. An exact test for randomness of mating. *J. Genet*. **52**, 631-635 (1954).
5. Robertson, A., & Hill, H.G. Deviations from Hardy-Weinberg proportions: sampling variances and use in estimation of inbreeding coefficients. *Genetics* 107: 703-718 (1984).
6. Cockerham, C.C. & Weir, B.S. Digenic descent measures for finite populations. *Genet. Res*. **30**, 121-147 (1977).
7. Ross, M.G. et al. Characterizing and measuring bias in sequence data. *Genom. Biol.* **14**, R51 (2013).
8. Haak W. et al. Massive migration from the steppe was a source for Indo-European languages in Europe. *Nature* **522**, 207–211 (2015).
9. Takahata, N. Gene identity and genetic differentiation of populations in the finite island model. *Genetics.* **104**, 497-512 (1983).
